# Discovery of novel benzylquinazoline molecules as p97/VCP inhibitors

**DOI:** 10.3389/fphar.2023.1209060

**Published:** 2023-06-14

**Authors:** Xiaoyi Zhang, Lingna Jiang, Yixin Li, Qiqi Feng, Xiulin Sun, Yaonan Wang, Ming Zhao

**Affiliations:** ^1^ Department of Medicinal Chemistry, School of Pharmaceutical Sciences, Capital Medical University, Beijing, China; ^2^ Beijing Area Major Laboratory of Peptide and Small Molecular Drugs, Engineering Research Center of Endogenous Prophylactic of Ministry of Education of China, Beijing Laboratory of Biomedical Materials, Beijing, China; ^3^ Core Facilities Centre, Capital Medical University, Beijing, China

**Keywords:** p97/VCP inhibitor, ATPase activity, organic synthesis, antiproliferative and anti-tumor activity, ubiquitin–proteasome system

## Abstract

**Introduction:** Protein p97 is an extensively investigated AAA ATPase with various cellular activities, including cell cycle control, ubiquitin–proteasome system, autophagy, and NF-κB activation.

**Method:** In this study, we designed, synthesized and evaluated eight novel DBeQanalogs as potential p97 inhibitors *in vivo* and *in vitro*.

**Results:** In the p97 ATPase inhibition assay, compounds **6** and **7** showed higher potency than the known p97 inhibitors, DBeQ and CB-5083. Compounds **4-6** dramatically induced G0/G1 phase arrest in the HCT116 cells, and compound **7** arrested the cells in both G0/G1 and S phases. Western blots showed elevated levels of SQSTM/p62, ATF-4, and NF-κB in HCT116 cells with the treatment of compounds **4–7**, confirming their role in inhibiting the p97 signaling pathway in cells. In addition, the IC_50_ of compounds **4–6** against HCT116, RPMI-8226, and s180 proliferation were 0.24–6.9 µM with comparable potency as DBeQ. However, compounds **4–6** displayed low toxicity against the normal human colon cell line. Thus, compounds **6** and **7** were proved to be potential p97 inhibitors with less cytotoxicity. *In vivo* studies using the s180 xenograft model have demonstrated that compound **6** inhibited tumor growth, led to a significant reduction of p97 concentration in the serum and tumor, and indicated non-toxicity on the body weight and organ-to-brain weight ratios except for the spleen at the dose of 90 μmol/kg/day for 10 days. Furthermore, the present study indicated that compound **6** may not induce s180 mice myelosuppression often observed in the p97 inhibitors.

**Conclusion:** Compound **6** displayed high binding affinity to p97, great p97 ATPase inhibition, selective cytotoxicity, remarkable anti-tumor effect, and upregulated safety, which improved the clinical potential of p97 inhibitors.

## Graphical Abstract

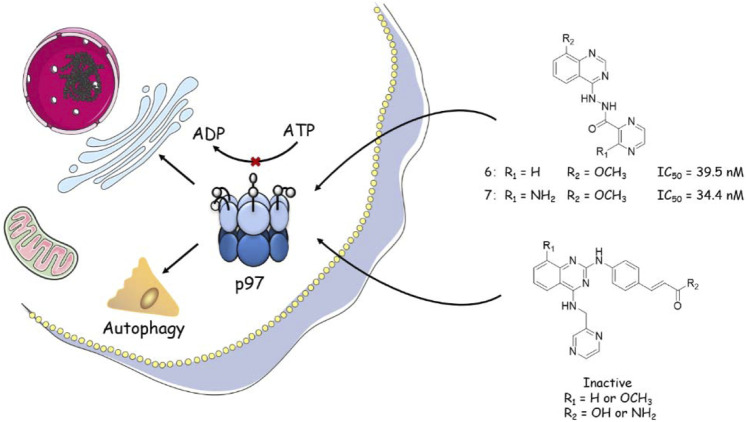

## 1 Introduction

Members of the protein AAA^+^ family (ATPases associated with diverse cellular activities) act as molecular chaperones in a large number of cellular events, using energy generated from ATP hydrolysis to remodel the structure of substrate proteins. p97, also called VCP in animals or Cdc48 in yeast, is an extensively studied member of the AAA family and is ubiquitous in all kinds of cells. Structurally, p97 is a hexameric ring, with each protomer comprising an N-terminal domain, two AAA domains (D1 and D2), and a C-terminal domain, all joined by short, well-conserved linkers (Chou et al., 2014; Xia et al., 2016; Costantini et al., 2021). Like other AAA proteins, p97 plays a critical role in a range of cellular activities, including protein quality and cell cycle control (Wang et al., 2021), ubiquitin–proteasome system (Nakkas et al., 2021), transcriptional activation (Madigan et al., 2019), DNA damage repair (Swan et al., 2021), autophagy (Yao et al., 2021), membrane fusion (Escobar-Henriques and Anton, 2020), and nuclear factor kappa-light-chain-enhancer of activated B-cell (NF-kB) activation (Schweitzer et al., 2016). The remarkable functional diversity of p97 is mainly because of its mediation in protein unfolding and complex disassembly through the ubiquitin–proteasome degradation pathway, which is fundamental in protein catabolism (Stolz et al., 2011). p97 also has a key role in protein homeostasis, as evidenced by its association with various diseases (van den Boom and Meyer, 2018; Lan et al., 2017). Several lines of clinical evidence suggest that the expression levels of p97 are upregulated in many cancers, such as melanomas, thyroid follicular cancer, esophageal carcinoma, gastric carcinoma, pancreatic cancer, breast cancer, osteosarcoma, and lung cancer (Huryn et al., 2020). Therefore, p97 is also implicated as a target of cancer therapeutics.

**FIGURE 1:**
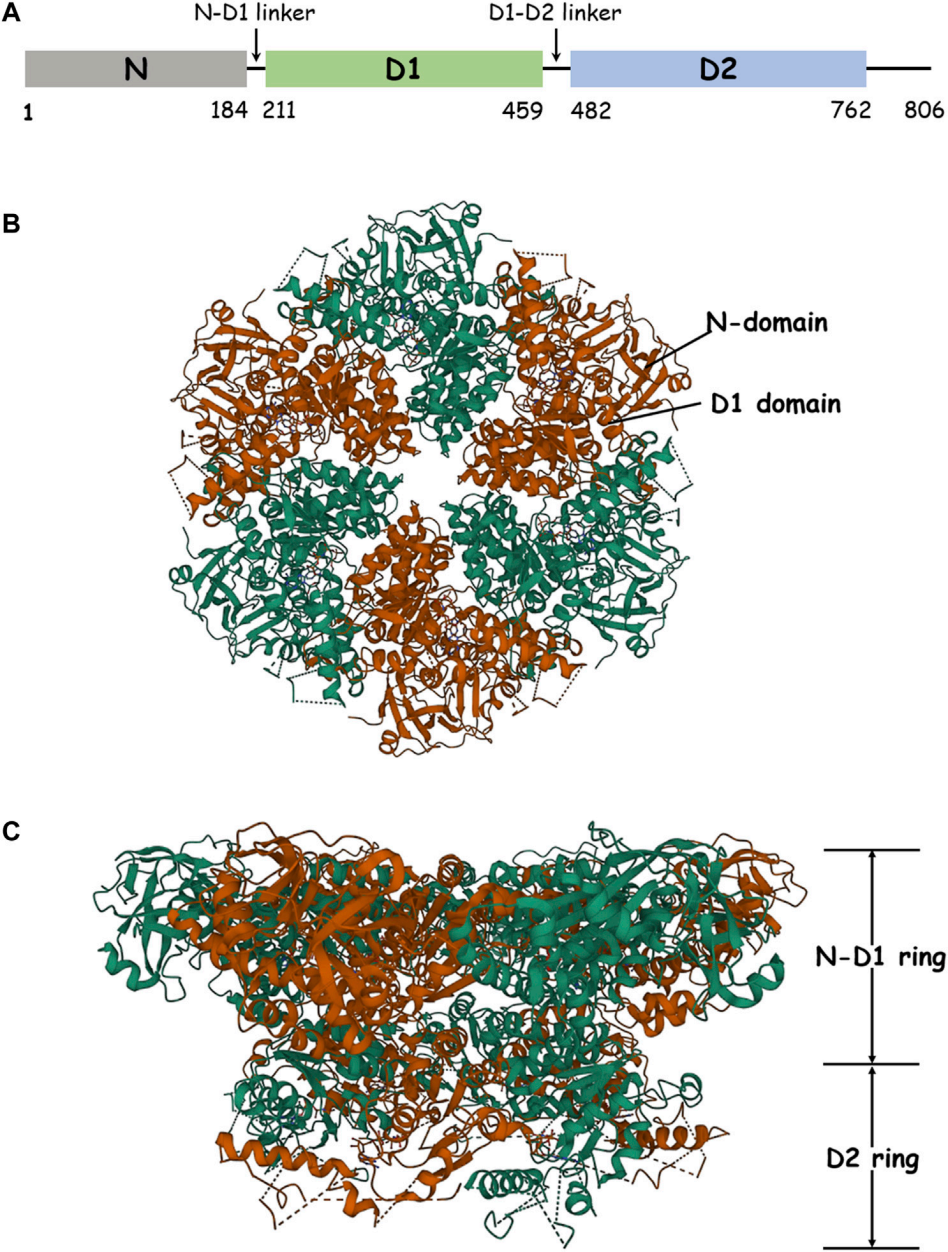
Structure of p97. **(A)** Schematic domain organization of p97. **(B)** The structure of hexameric p97 (PDB: 3CF2) is viewed down the sixfold symmetry axis showing the N–D1 ring. The D1 domain and the N-domain are indicated with arrows and labeled for one of the six subunits. **(C)** The side view of p97 is presented.

**FIGURE 2:**
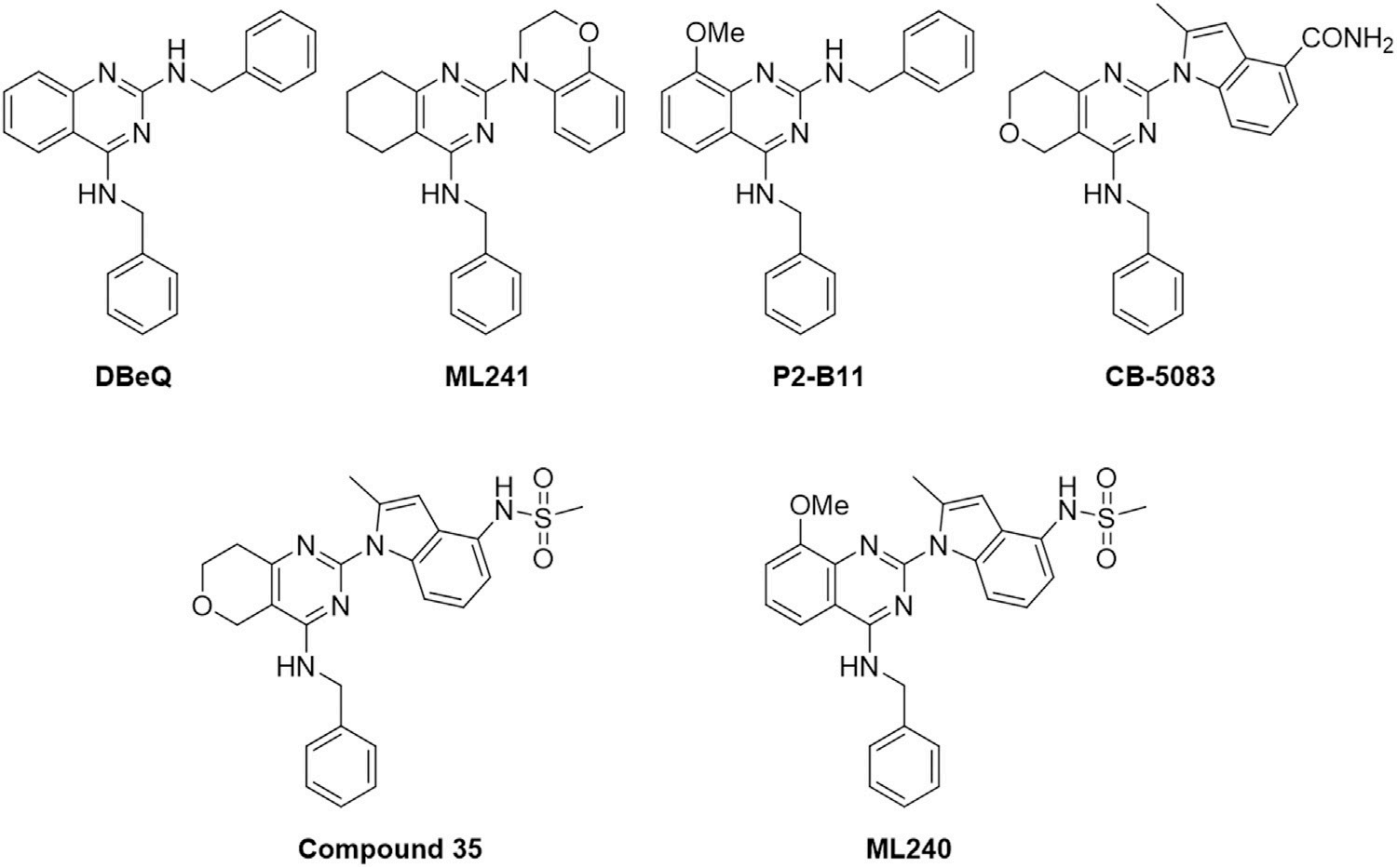
Structures of known p97 inhibitors.

Figure 1 outlines the examples of the latest p97 inhibitors developed. DBeQ, which emerged from a high-throughput screening at The Scripps Research Institute, is identified as a selective, potent, reversible, and ATP-competitive p97 inhibitor and mainly targets the D2 ATP site. DBeQ decreases autophagy, ubiquitin-dependent protein degradation, and endoplasmic reticulum-associated degradation (ERAD) networks (Chou et al., 2011). This compound has also been identified as a selective inducer of apoptosis in multiple myeloma, cervical carcinoma, and renal carcinoma compared to normal human fetal lung fibroblasts (Chou and Deshaies, 2011). SAR studies of this scaffold demonstrated that benzylamine was the major pharmacophore maintaining the inhibitory activity against p97. Therefore, efforts to improve potency have focused on the quinazoline backbone. On the basis of these attempts, many DBeQ derivatives, such as ML-240 (IC_50_ = 0.11 µM), ML-241 (IC_50_ = 0.1 µM), P2-B11 (IC_50_ = 3.4 µM), compound 35 (IC_50_ = 0.036 µM), and CB-5083 (IC_50_ = 0.011 µM), have been developed by several research groups (Figure 2). ML-240 and ML-241 inhibited the degradation of a p97-dependent but not a p97-independent proteasome substrate and also impaired the ERAD pathway (Fang et al., 2015; Wang et al., 2019). Further characterization revealed that ML-240 had a broad antiproliferative activity through NCI 60-cancer-cell-line screening but relatively lower activity toward normal cells. In 2019, the co-crystallized structure of CB-5083 and N-terminal domain-truncated p97 was elucidated, which provided an atomic basis for the specialness of CB-5083 toward the D2 domain (Tang et al., 2019). *In vitro*, treatment of tumor cells (multiple myeloma, B acute lymphoblastic leukemia in particular) with CB-5083 leads to cytotoxicity, cell cycle arrest, apoptosis, and accumulation of polyubiquitinated proteins and retention of ERAD substrates (Anderson et al., 2015; Le Moigne et al., 2017; Wang et al., 2019; Zhao et al., 2020). *In vivo*, CB-5083 demonstrated significant tumor growth inhibition in solid tumor and hematological xenograft models (Zhou et al., 2015). Based on the promising preclinical data, CB-5083 was evaluated in two phase 1 clinical studies as a selective p97 inhibitor, indicated for advanced solid tumors (NCT02243917 (ClinicalTrials, 2018a)) and lymphoid hematological malignancies (NCT02223598 (ClinicalTrials, 2018b)). However, these clinical trials were terminated as a result of adverse effects on vision loss in patients resulting from an off-target inhibition of phosphodiesterase-6 (PDE6) (Leinonen et al., 2021). Thus, modifying the DBeQ-based p97 inhibitor to prevent potential off-target effects has attracted much attention from researchers. Herein, we report a medicinal chemistry study set up to obtain novel p97 inhibitors and investigate the p97 pathway.

## 2 Results and discussion

### 2.1 Structure-based drug design to develop inhibitors of p97

The initial discovery of the parent compound, DBeQ (dibenzylquinazoline), was originally developed at The Scripps Research Institute, part of the NIH Roadmap initiative. DBeQ was identified as a reversible, ATP-competitive p97 inhibitor that showed an IC_50_ value of <10 μM in the ATPase activity assays (Chou et al., 2011). Hit-to-lead optimization efforts resulted in the discovery of some analogs, ML240, ML241, P2-B11, and compound **35** (Chou et al., 2013; Fang et al., 2015; Wang et al., 2019). Although these compounds were valuable research tools, their potency and pharmaceutical properties were insufficient to determine the impact of p97 inhibition *in vivo*. Herein, we report our lead optimization efforts and SAR analysis. To obtain new p97 inhibitors, we selected the DBeQ as the lead because of its potency and specificity, which are expected to bind into the D2 ATP-binding site. A previous computational docking study performed on DBeQ suggested that the phenyl group at the N4 position projects into a narrow cavity that is normally occupied by the purine ring of ATP, while only minor structural changes could be tolerated on the phenyl ring (Zhou et al., 2015; Ding et al., 2019).

In Group I, we replaced the phenyl ring with the pyrazine and chose the hydrazine group to substitute the secondary amino group at the N4 position to explore the effect of a longer spacer. Then, the benzamido group at the N2 position was eliminated to reduce the steric hindrance. In compounds **5** and **7**, we introduced NH_2_ on the pyrazine that would react with active-site residues. In compounds **6** and **7**, we added a methoxy group that has been well exploited in the design of kinase inhibitors (Egert-Schmidt et al., 2010; Min et al., 2016; Leitão et al., 2021). In Group II, the phenyl ring at the N4 position was replaced with the pyrazine with the same spacer length. The benzyl group at the N2 position was replaced with a cinnamic acid or a cinnamic amide, which was screened out by HTS (Figuerola-Conchas et al., 2020), leading to **10a–b** and **11a–b**. Compounds **10b** and **11b**, based on compounds **6** and **7**, featured a methoxy group.

### 2.2 Synthesis of compounds **4–7, 10, and 11**

**SCHEME 1:**
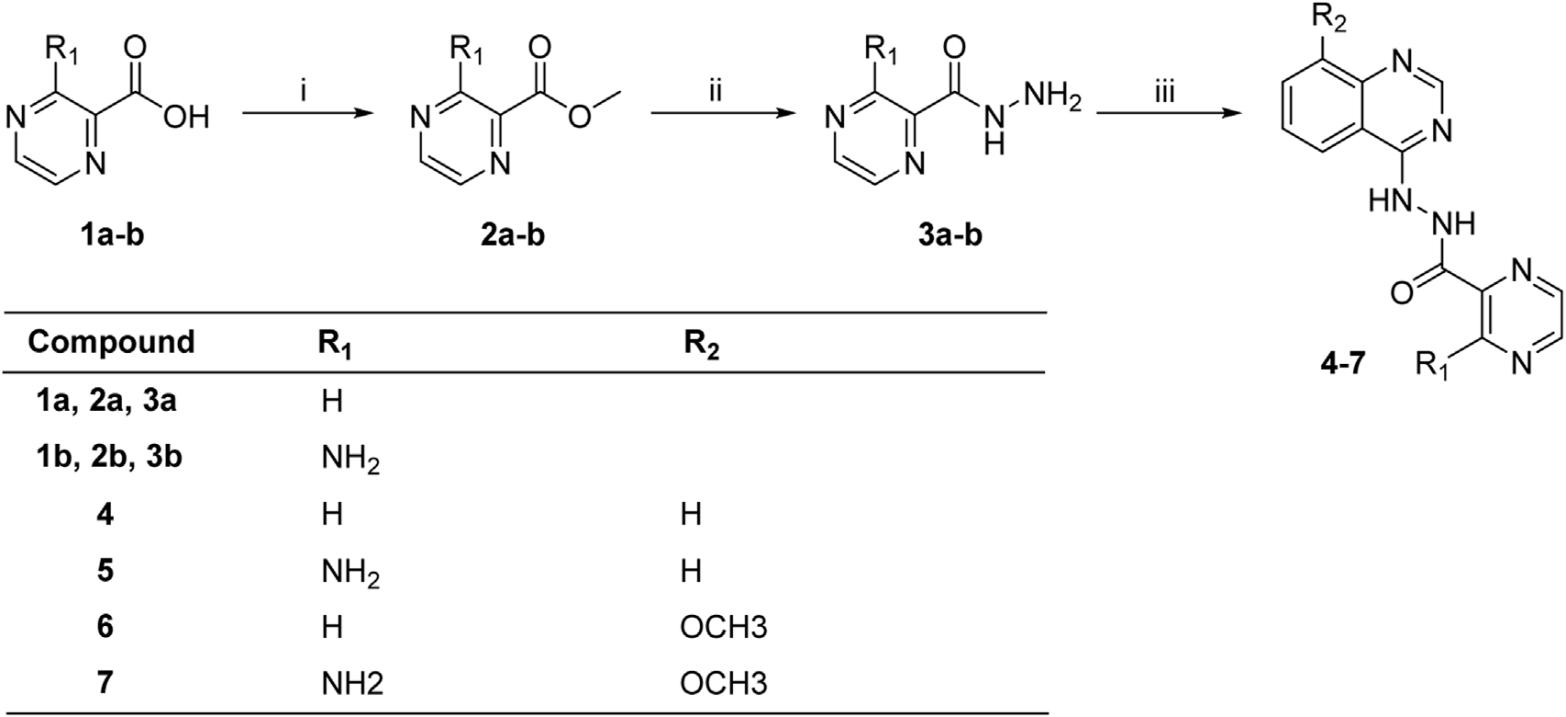
Synthesis of compounds **4–7**. Reagents and conditions: (i) SOCl_2_, CH_3_OH, 0°C to r.t., 24 h; (ii) 85% hydrazine hydrate, ethanol, 0°C to r.t., 4 h; (iii) 4-chloroquinazoline or 4-chloro-8-methoxyquinazoline, triethylamine, CH_3_CN, or DMF.

The synthesis of target molecules (**4–7**) was summarized in Scheme 1 **.** The pyrazine carboxylic acid **1a–b** were esterified to form **2a–b** containing methyl esters. Then, compounds **2a–b** were reacted with 85% hydrazine hydrate in the ethanol to produce key intermediates **3a–b** (81.1% and 24.0%, respectively). 4-Chloroquinazoline or 4-chloro-8-methoxyquinazoline was coupled with intermediates **3a–b** to yield compounds **4–7** (54.5%, 57.7%, 44.2%, and 46.6%, respectively).

**SCHEME 2:**
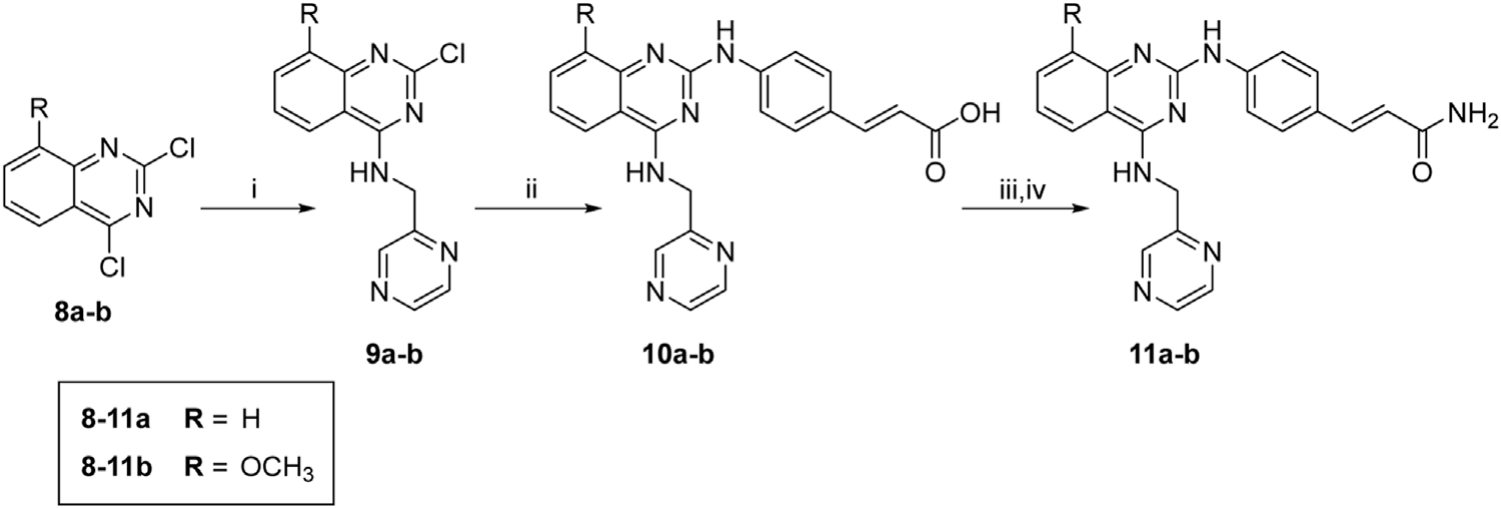
Synthesis of compounds **10a**, **11a**, **10b**, and **11b**. Reagents and conditions: (i) 1-(pyrazin-2-yl)methanamine hydrochloride, CH_3_CN, 0°C to r. t., overnight; (ii) (E)-4-aminocinnamic acid, XPhos, Pd_2_(dba)_3_, Cs_2_CO_3_, reflux, 48 h; (iii) SOCl_2_, 4 h; (iv) concentrated ammonia solution, THF, overnight.

The synthesis of target molecules (**11a–b**) is outlined in Scheme 2 **.** The 4-chloro group of **8a–b** was selectively substituted with 1-(pyrazin-2-yl)methanamine to yield intermediates **9a–b** (74.4%, 70.6%, respectively), which was coupled with 4-aminocinnamic acid in the presence of Pd_2_(dba)_3_, X-Phos, and cesium carbonate to give compounds **10a–b** (47.2% and 43.4%, respectively). Then, **10a–b** were ammonified with concentrated ammonia solution to produce **11a–b** (30.6% and 42.1%, respectively).

### 2.3 Determining p97 binding affinity of compounds **4–7**, **10a–b**, and **11a–b**

**FIGURE 3:**
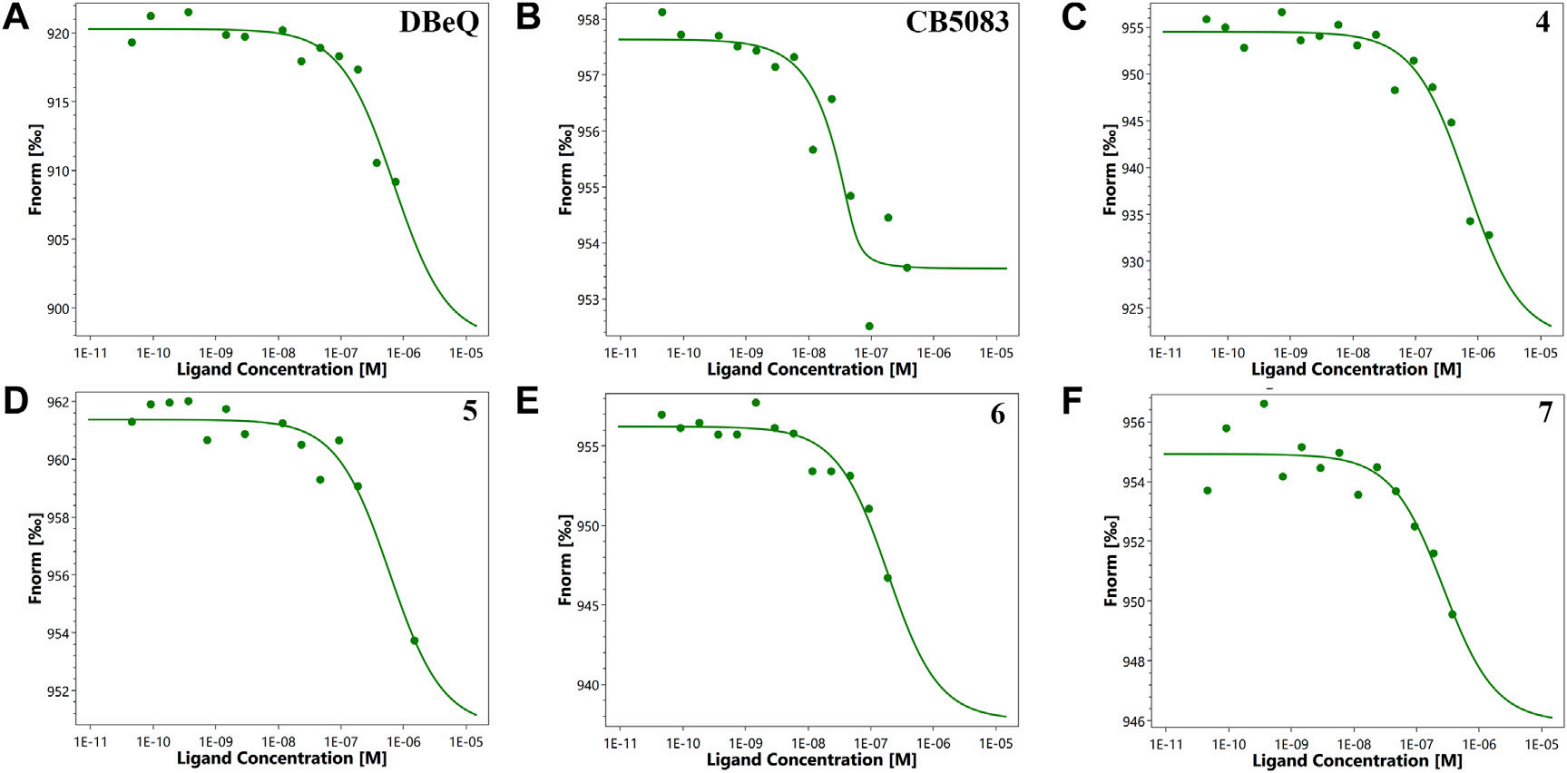
Microscale thermophoresis measurements of the binding affinity of DBeQ **(A)**, CB-5083 **(B)**, and compounds **4–7 (C–F)** for p97. Fnorm (normalized fluorescence) = Fhot/Fcold.

First of all, the binding affinity between p97 and tested compounds was measured with fluorescent-labeled His6-p97 through a microscale thermophoresis (MST) assay, which represented a direct interaction between the protein and a ligand. As shown in Table 1, DBeQ and CB-5083 served as positive controls, with *K*_d_s (dissociation constant) of 0.68 and 0.0017 µM, respectively. Among the eight tested compounds, Group I compounds **4**–**7** exhibited lower *K*_d_ values ranging from 0.14 to 0.64 μM compared with DBeQ, indicating that the absence of a 5-position substituent on quinazoline did not affect the binding efficiency toward p97. In particular, compounds **6** and **7** with a methyloxy functional group at the 8-position of the quinazoline ring showed more than threefold higher binding affinity to p97 than DBeQ. Although Group II compounds (**10a**–**b** and **11a**–**b**) showed very low binding affinity, *K*_d_ = 5.0 μM for **10a** and *K*_d_ > 10 μM for **10b** and **11a**–**b**, suggesting that the acrylic acid and acrylamide functional groups were not conducive to the binding with p97. The MST binding curves of **4**–**7** are displayed in Figure 3, and the curves for other compounds are provided in the Supplementary Material.

**TABLE 1:** Dissociation constant (*K*_d_) of target compounds with p97 and the IC_50_ values (nM) of inactivation activities against p97.

Compound	*K*_d_ (μM)	IC_50_ (nM)
DBeQ	0.68 ± 0.059	657.4
CB-5083	0.0017 ± 0.00088	57.6
**4**	0.64 ± 0.26	108
**5**	0.40 ± 0.15	72
**6**	0.14 ± 0.36	39.5
**7**	0.18 ± 0.061	34.4
**10a**	5.0	Inactive
**10b**	>10	Inactive
**11a**	>10	Inactive
**11b**	>10	Inactive

### 2.4 Inhibition of p97 ATPase activity by compounds **4–7**, **10a–b**, and **11a–b**

**FIGURE 4:**
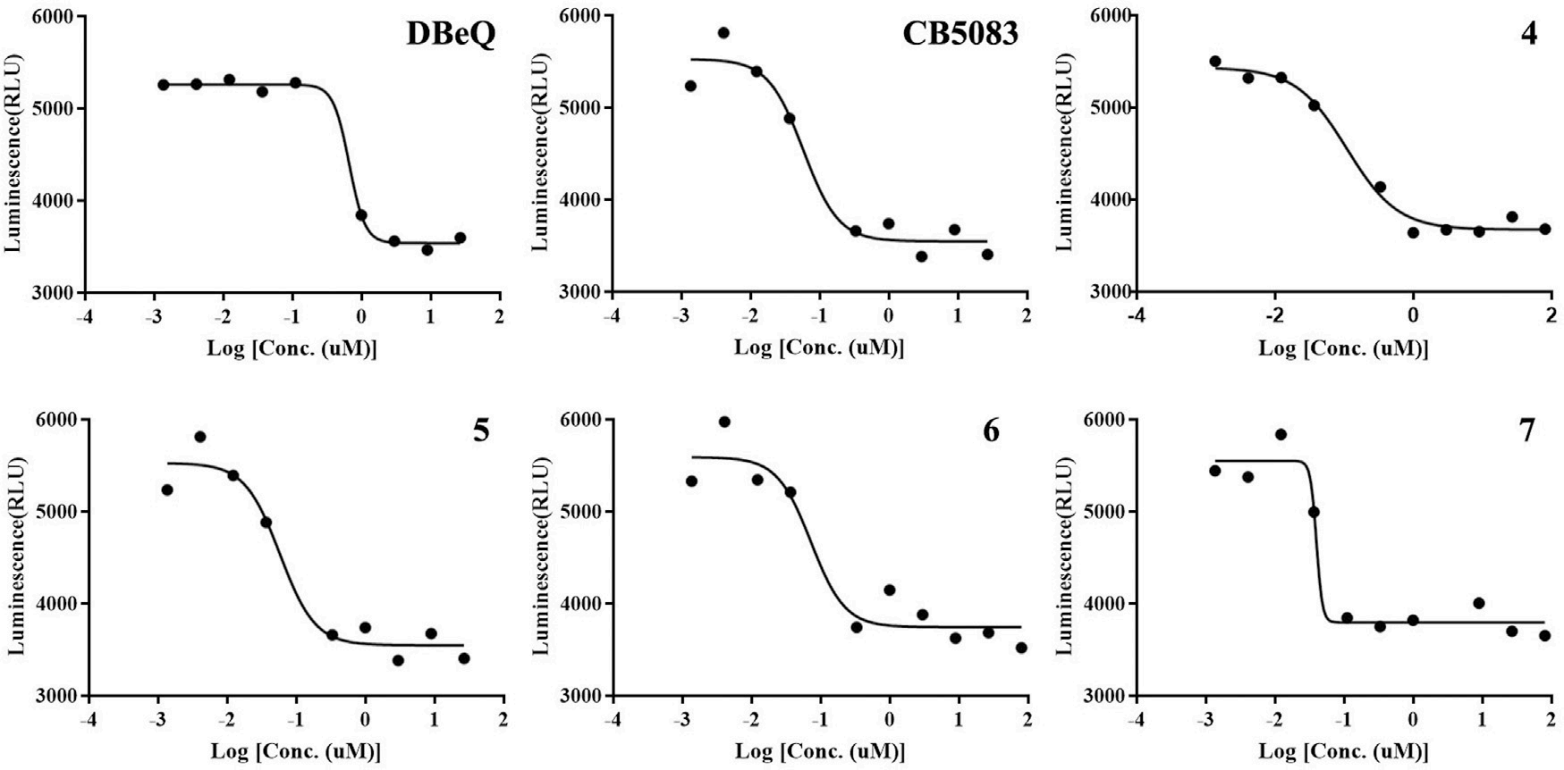
Inhibition of p97 ATPase activity upon exposure to the increasing amount of compounds. Titration curves of the *in vitro* ATPase assays for DBeQ, CB5083, and compounds **4–7** inhibition of p97.

As the major indicator of p97 inhibitors, the inactivation of p97 ATPase by the tested compounds was determined using an ADP-Glo kinase assay, in which the formation of ADP represented the activity of ATPase. The inhibition curves (luminescence vs. log [I]) of tested compounds and positive controls (DBeQ and CB-5083) are presented in Figure 4, and their IC_50_s are listed in Table 1. Obviously, the IC_50_ values of DBeQ and compounds **4–7** in ATPase inhibition are basically consistent with their p97 binding affinities (*K*_d_s), suggesting that DBeQ and **4–7** probably act on the same active cavity of p97, the so-called D2 domain. Interestingly, CB-5083 exhibited an extremely high binding affinity (*K*_d_ = 0.0017 µM) to p97, but its inhibitory activity on p97 ATPase was not as strong as compounds **6** and **7**. This could be explained by the ineffective binding of CB-5083 to p97, which contributes to its off-target actions on other targets, inducing serious side effects in the clinical trial (Leinonen et al., 2021). The same as the MST assay, **10a–b** and **11a–b** showed very weak inhibition of p97 ATPase.

### 2.5 Antiproliferative effect of compounds **4–7** in various cell lines

Cytotoxicity of compounds **4–7** was evaluated in five human cancer cell lines (A549, HCT116, K562, HepG2, and RPMI-8226), a mouse cancer cell line (s180), and a normal human colon mucosal epithelial cell line (NCM460) with the MTT method. Cells were treated with the tested compounds in a variety of concentrations and incubated at 37°C for 48 h before measurement. IC_50_ values were calculated using the percentage of growth of treated cells *versus* the blank control (Table 2). The results showed that CB-5083 showed the most potent antiproliferative activity. The potency of compounds **4–6** varied in different types of cancer cells. The IC_50_ of compounds **4–6** against HCT116, RPMI-8226, and s180 were 0.24–6.9 µM. Compounds **4–6** had comparable potency as DBeQ. Furthermore, we evaluated all compounds’ potency toward the normal human cell line NCM460. Compounds **4–6** were 21.1–47.7-fold less effective in NCM460, whereas DBeQ exhibited a similar effect. However, CB5083 was 9.87–1.92 times more effective than NCM460. Therefore, compounds **4–6** displayed low toxicity against the normal human colon cell line, with selectivity indices greater than six across the carcinoma cells, indicating that the compounds possess potential in the development of low-toxicity chemotherapeutic agents.

**TABLE 2:** Inhibition of cell proliferation by tested compounds **4–7**.

Compound	IC_50_			(µM)			
	A549	HCT116	K562	HepG2	RPMI-8226	s180	NCM460
DBeQ	11.63 ± 0.84	4.78 ± 0.22	5.64 ± 1.08	7.57 ± 1.20	4.29 ± 2.46	2.69 ± 0.82	6.44 ± 0.36
CB-5083	0.77 ± 0.38	0.47 ± 0.16	0.70 ± 0.42	0.16 ± 0.03	0.15 ± 0.02	0.24 ± 0.29	0.078 ± 0.015
**4**	>100	3.48 ± 0.21	66.65 ± 9.97	>100	5.24 ± 0.45	1.043 ± 0.37	49.77 ± 3.25
**5**	24.25 ± 5.39	5.83 ± 1.03	1.79 ± 0.73	4.53 ± 1.26	0.75 ± 0.01	6.9 ± 2.00	24.17 ± 1.05
**6**	>100	2.94 ± 0.10	>100	>100	3.61 ± 0.27	1.72 ± 0.69	36.23 ± 2.89
**7**	>100	>100	>100	>100	39.84 ± 0.95	32.35 ± 2.57	27.33 ± 1.62

### 2.6 Compounds **4–7** inducing apoptosis and cell cycle arrest in HCT116 cells

**FIGURE 5:**
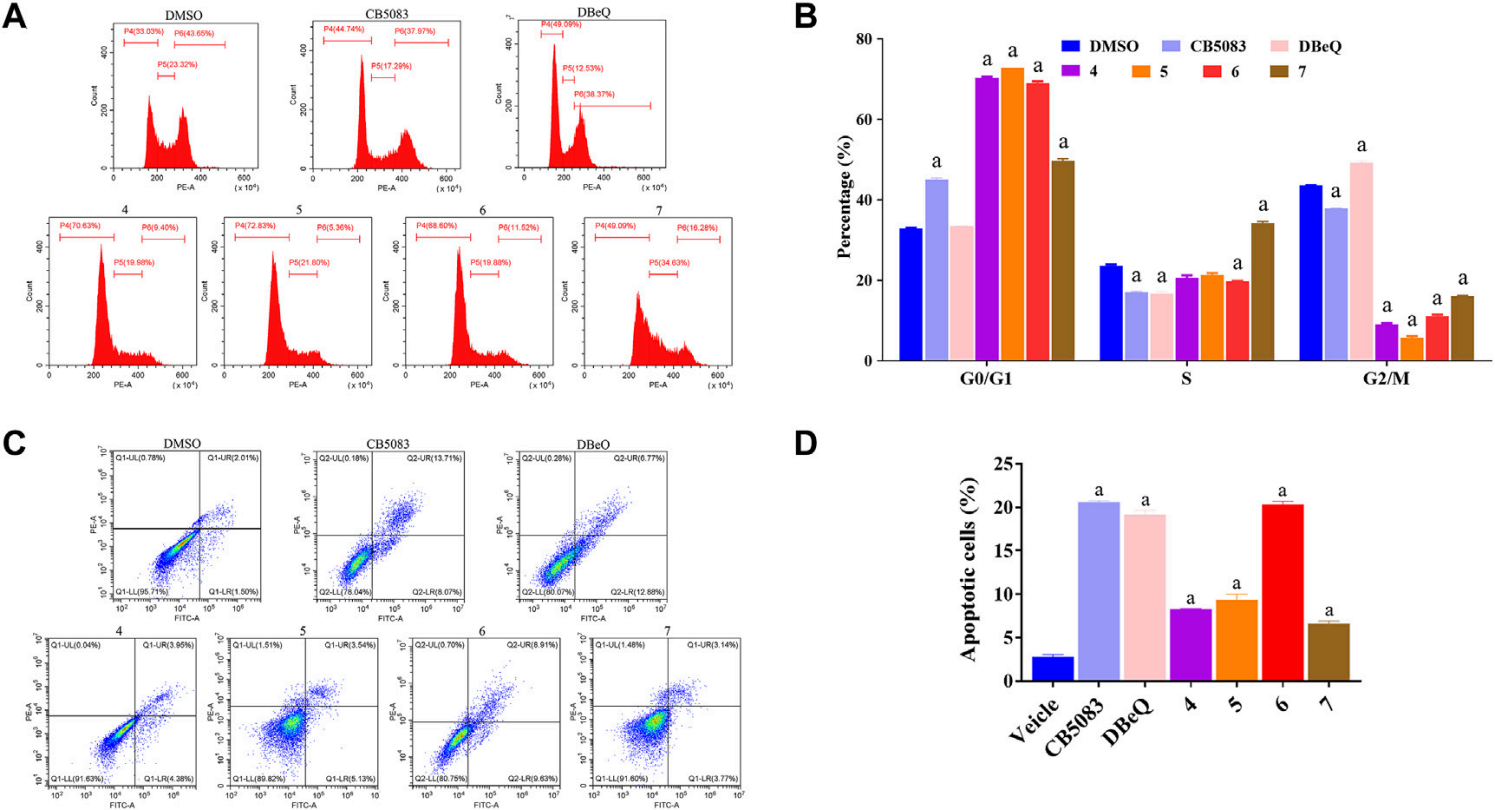
Cell cycle and apoptosis assays of HCT116 cells treated with CB5083, DBeQ, and compounds **4–7**. **(A)** Cell cycle assay of HCT116 cells treated with CB5083 (20 μM), DBeQ (20 μM), and compounds **4–7** (20 μM). The cells were treated for 12 h, and cell cycle assays were performed using PI staining and flow cytometry. **(B)** Lengths of the different cell cycle phases with CB5083, DBeQ, and compounds **4–7**-treated HCT116 cells. a, *p* < 0.001 compared with the control group. **(C)** Apoptosis assay of HCT 116 cells treated with CB5083, DBeQ, and compounds **4–7**. The cells were treated for 12 h, and cell apoptosis was detected by annexin V/PI staining and flow cytometry. **(D)** Percentages of apoptotic HCT116 cells after CB5083, DBeQ, and compounds **4–7** treatment. a, *p* < 0.001 compared with the control group.

Recent evidence suggested that p97 inhibitors could induce apoptosis and inhibit the cell cycle in cancer cells (Zhao et al., 2020; Desdicioglu et al., 2021). Thus, we evaluated the rates of apoptosis at 12 h after treatment with 10 μM of DBeQ (positive control), CB5083 (positive control), or compounds **4–7**. Apoptosis was measured by flow cytometry after staining with annexin V and propidium iodide (PI) in HCT116 (Figure 5). Annexin V and PI exhibited the percentage of early apoptotic cells (annexin V+ and PI−, quadrant Q1) and late apoptotic cells and necrosis cells (annexin V+ and PI+, quadrant Q2). DBeQ and CB5083 induced apoptosis in HCT116 cells, and the proportion of apoptotic cells was significantly higher (DBeQ: 19.65%; CB5083: 20.42%) than control cells (3.5%, *p* < 0.001). A similar trend was also found for compound **6**, with a 20.68% apoptotic proportion. However, compounds **4**, **5**, and **7** led to a lower degree (**4**: 8.31%; **5**: 9.34%; and **7**: 8.94%).

We also investigated the effects of compounds **4–7** on the cell cycle at 12 h at the same concentration as apoptosis measurement. Compared to the vehicle, a significantly higher number of cells with 2 N DNA content (diploid) were noted in compounds **4–7** treated groups, suggesting that cells already in the G0/G1 phase were not able to continue to the S and G2/M phases (compounds 4–7:44.92%–70.35%; vehicle: 32.82%; *p* < 0.01). Interestingly, the cell proportion in the S phase in the compound **4**-treated group was notably higher than that in the vehicle group. The DBeQ group showed a G0/G1 phase fraction of 33.53%, not significantly different from the vehicle. However, the percentage of cells in the S phase decreased from 23.61% to 16.74%, and the percentage of cells in the G2/M phase increased from 43.51% to 49.30%, respectively. This result indicated that DBeQ arrested the cell cycle at the G2/M phase in HCT116 cells. Therefore, the effects of compounds **4–7** on the cell cycle were completely different from the known p97 inhibitor DBeQ.

### 2.7 Compounds **4–7** regulated the ER-associated degradation (ERAD), autophagy, and NF-κB activation pathways in HCT116 cells

To provide more insights into the impact of test compounds on the p97 pathway, we investigated the effects of compounds **4–7** on p97 downstream biomarkers in ER-ERAD, autophagy, and NF-κB activation pathways. ATF4 is an activating transcription factor potentially implicated in the irresolvable ER stress-triggered cell death process (Bastola et al., 2016; Gugliotta et al., 2017). Protein p62 is an autophagy adaptor that binds to aggregated proteins such as ubiquitin, forming autophagosomes (Desdicioglu et al., 2021). When autophagy is activated, the p62 protein is degraded like a substrate to indicate the progression of autophagy (Liu et al., 2016). An elevated p97 level leads to increased proteasomal degradation of IκB, which is responsible for the NF-κB-induced pro-survival signaling (Valle et al., 2011).

**FIGURE 6:**
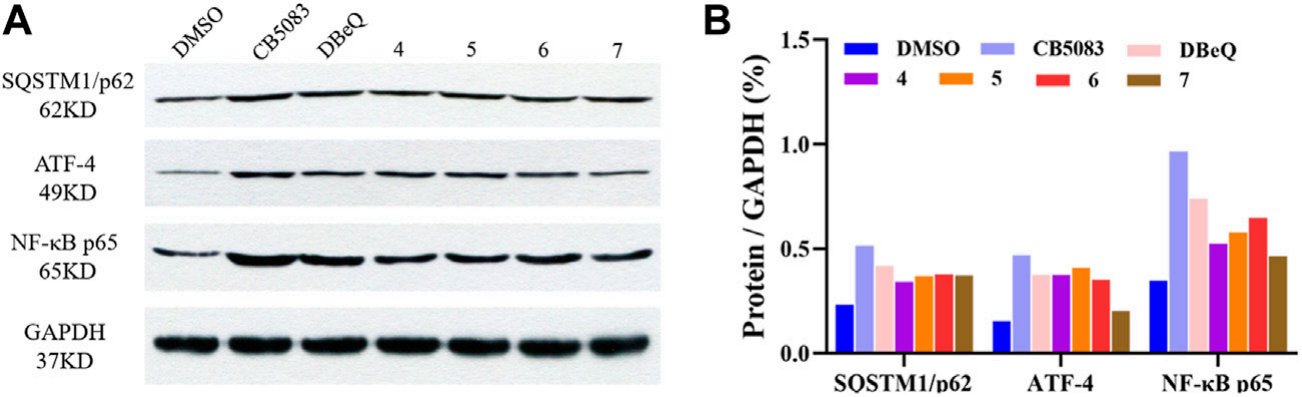
Changes in biomarkers of p97 inhibition. **(A)** Western blot analysis following treatment with DBeQ, CB5083, and compounds **4–7** in HCT116 cell lines for 8 h. **(B)** The relative expression level was normalized with the internal reference GAPDH and shown in columns.

It was found that the positive control DBeQ (10 μM) and CB-5083 (0.5 μM) exhibited increased levels of the ER stress biomarker (ATF4) and induced accumulation of the autophagosome formation in the cytoplasm after 8 h treatment. The observed protein expression of NF-κB p65 was promoted in cells, which was also correlated with the p97 inhibition. The tested compounds **4–7** caused similar induction of SQSTM1/p62, ATF4, and NF-κB, which indicates their action on the p97 signaling pathway (Figure 6).

#### 2.7.1 Pharmacological evaluation

**FIGURE 7:**
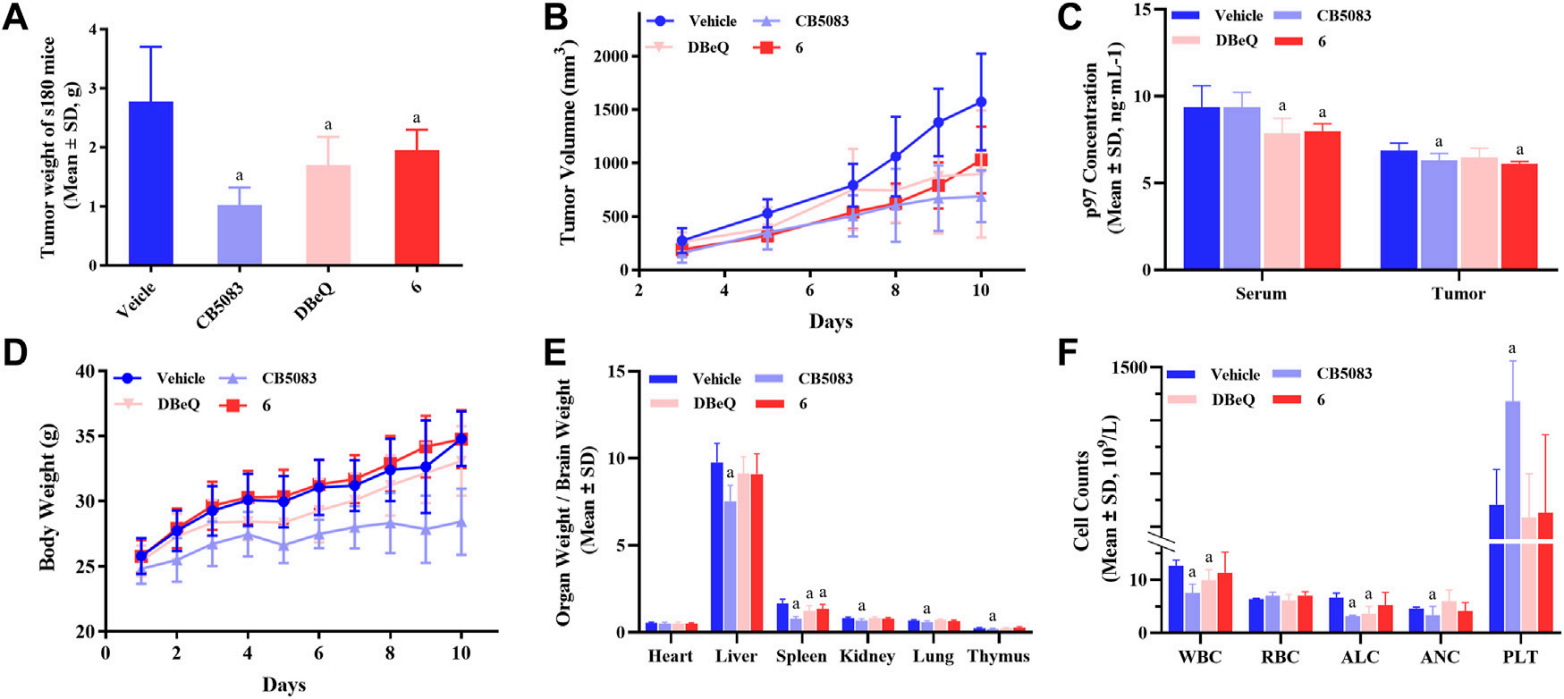
Inhibition of p97 suppresses tumor growth and preliminary safety evaluation. To implant subcutaneous tumors, 0.2 ml of NS containing 2 × 10^6^ viable tumor cells was injected under the skin at the right armpit of ICR mice. After 24 h, mice were randomly divided into seven groups and treated for 10 consecutive days, i.e., compound **6** (oral administration: 90 μmol/kg/day), DBeQ and CB5083 (oral administration: 90 μmol/kg/day, positive control), and 0.5% CMC-Na (oral administration, vehicle control). **(A)** Tumor weights of s180 mice treated with compounds (*n* = 12), a, compared to vehicle, *p* < 0.05; b, compared to DBeQ, *p* > 0.05. **(B)** Growth of tumors formed by s180 cells and treated with compounds or vehicle (*n* = 12). **(C)** p97 expression measurement in mice serum and tumor by ELISA (*n* = 12). **(D)** Body weight change of s180 mice after oral administration (*n* = 6), a, compared to vehicle, *p* < 0.05. **(E)** Organ-to-brain weight ratio was calculated by comparing organ weight with mice brain weight on day 10 (*n* = 12), a, compared to vehicle, *p* < 0.05. **(F)** WBC, RBC, PLT count, ALC, and ANC obtained from s180 10 days after treatment with compounds (*n* = 3), a, compared to vehicle, *p* < 0.05.

In order to evaluate the *in vivo* activity, compound **6** was further investigated for the anti-tumor activity in the s180 mouse xenograft model due to its good p97 inhibition activity and cytotoxicity. DBeQ and CB5083 were selected as the positive controls. Compound **6**, DBeQ, and CB5083 were administered orally at the dose of 90 μmol/kg/day as a suspension in 0.7% of CMC-Na aqueous solution every day for 10 days. Treatment with CB5083, DBeQ, and compound **6** inhibited tumor progression (inhibition 29.6%–63.18%) to an extent compared favorably (*p* < 0.05) to the vehicle (Figures 7A, B). Compound **6** demonstrated a similar level of efficacy with DBeQ.

On day 10, compound **6** treatment led to a significant reduction of p97 concentration in the serum and tumor. However, DBeQ treatment simply lowered the p97 concentration in the serum, and CB5083 treatment only lowered the p97 concentration in the tumor (Figure 7C).

The changes in body weight in mice are shown in Figure 7D. No obvious loss of body weight was observed with compound **6** treatment, and no mice died during the *in vivo* experiments, indicating non-toxicity of compound **6** on the body weight at the dose of 90 μmol/kg/day. Instead, CB5083 exhibited obvious weight loss effects in mice at the same dose, and death occurred on day 8.

To assess the sub-chronic toxicity, we compared the organ-to-brain weight ratio of various organs between compound and vehicle mice. Figure 7E shows that mice treated with 90 μmol/kg/day CB5083 exhibited significant differences (*p* < 0.05) in organ-to-brain weight ratios for the liver, kidney, lung, and thymus compared with the vehicle group. Significantly, the decreased spleen-to-brain weight ratio was observed in mice treated with compound **6** (*p* < 0.05), and the other organ-to-brain weight ratio showed no difference.

A major concern associated with CB5083 therapy of solid tumors and multiple myeloma is dose-limiting, chemotherapy-induced bone marrow toxicity. To determine the effects on myelosuppression, WBC, RBC, PLT count, ALC, and ANC were performed in peripheral blood samples of s180 mice (Figure 7F) on day 10. CB5083 produced significantly lower degrees of WBC, ALC, and ANC compared to vehicle treatments; however, the PLT count of the CB5083 group showed significantly higher changes than that of the vehicle group. These results indicated that CB5083 induced a reduction in peripheral blood cell counts of s180 mice and may cause myelotoxicity. In mice treated with DBeQ, WBC and ALC appeared to be significantly reduced; other tested data exhibited no significant alterations. The WBC, RBC, ALC, ANC, and PLT count showed no significant changes after the 10-day treatment of compounds **6**. We anticipate that compound **6** will be more tolerable than CB5083 or DBeQ and decrease myelosuppression often observed in chemotherapy.

## 3 Conclusion

To develop less toxic and more potent p97 inhibitors, we designed and synthesized eight novel DBeQ analogs, **4–7**, **10a–b,** and **11a–b**. The p97 binding assay and p97 ATPase inhibition assay indicated that analogs **4–7** exhibited higher potency than DBeQ, whereas compounds **6** and **7** were more potent than CB-5083 in the p97 ATPase inhibition assay. The following cytotoxic assay showed that compounds **4–7** were much less toxic than CB-5083 and DBeQ. Furthermore, compounds **4–6** dramatically induced G0/G1 phase arrest in the HCT116 cells, and compound **7** held the cells in both G0/G1 and S phases. However, DBeQ and CB-5083 obviously showed a very mild impact on the cell cycle, suggesting different mechanisms between the tested compounds and known p97 inhibitors in cell metabolism. Three downstream checkpoints of p97 (SQSTM/p62, ATF-4, and NF-kB) were tested to identify the inhibition of the p97 pathway in the cell. Compounds **4–7** showed elevated levels of SQSTM/p62, ATF-4, and NF-kB as DBeQ and CB-5083, which suggested suppression of p97 signaling, confirming their role as p97 inhibitors. The *in vivo* xenograft mice models of s180 cells showed that compound **6** effectively inhibited the tumor growth and reduced the p97 concentration in the serum and tumor, and almost no obvious side effects were observed. Therefore, compound **6** was found in the study to be more potent than known p97 inhibitors, DBeQ and CB-5083, and less toxic than the investigational drug CB-5083 *in vitro* and *in vivo*.

## 4 Experimental section

### 4.1 Chemistry

All reagents, solvents, and starting materials were purchased from Sigma-Aldrich. DBeQ and CB-5083 were purchased from MCE (Shanghai, China). ^1^H and ^13^C NMR spectra were recorded on a Bruker AMX-300 or a Bruker AMX-800 spectrometer, for which dimethylsulfoxide (DMSO-*d*_6_) was the solvent and tetramethylsilane was the internal standard. ESI mass spectra were recorded on a 9.4-T solariX Fourier transform ion cyclotron resonance mass spectrometer (Bruker Corp, Billerica, MA, United States). Chromatography was performed through Qingdao silica gel GF254 or Qingdao silica gel H60. HPLC was performed with Agilent Technologies 1200 Series HPLC system (Agilent Technologies, Santa Clara, CA, United States) on Eclipse XDB C18 column (5 μm, 4.6 mm × 150 mm). HPLC purity of each target compound was higher than 95%.

#### 4.1.1 Methyl pyrazine-2-carboxylate (**2a**)

To a solution of 48 ml of dry methanol was added SOCl_2_ (3.12 ml) dropwise at 0°C. After 30 min, 2-pyrazine carboxylic acid (**1a**, 1.5 g, 12.0 mmol) was added, and the reaction solution was warmed to room temperature and stirred for 24 h. The resulting solution was concentrated under vacuum. The product was precipitated by the addition of diethyl ether and collected by filtration to produce title compound **2a** (1.4 g, yield 84%) as a white solid. ESI^+^/MS: 139.0 [M + H]^+^. ^1^H NMR (300 MHz, DMSO-*d*_6_) δ 9.21 (s, 1H), 8.90 (d, *J* = 2.2 Hz, 1H), 8.82 (s, 1H), 3.93 (s, 3H) (Zhou et al., 2015).

#### 4.1.2 Pyrazine-2-carbohydrazide (**3a**)

To a solution of 580 mg of compound **2a** (4.2 mmol) in 10 ml of ethanol, 5 ml of 85% hydrazine hydrate solution was added at 0°C. The resulting solution was stirred at room temperature for 4 h. The precipitated white solids were filtered under vacuum and rinsed with dichloromethane (3 × 2 ml) and ethanol (3 × 2 mL) to yield the title compound **3a** (470 mg, yield 81%). ESI^+^/MS: 139.0 [M + H]^+^. ^1^H NMR (300 MHz, DMSO-*d*_6_) δ 10.13 (s, 1H), 9.13 (d, *J* = 1.5 Hz, 1H), 8.84 (d, *J* = 2.5 Hz, 1H), 8.70 (dd, *J* = 2.5, 1.5 Hz, 1H), 4.66 (s, 2H) (Zhou et al., 2015).

#### 4.1.3 Methyl 3-aminopyrazine-2-carboxylate (**2b**)

To a solution of 48 ml of dry methanol was added SOCl_2_ (3.12 ml) dropwise at 0°C. After 30 min, 3-aminopyrazine-2-carboxylic acid (**1b**, 1.84 g, 12.0 mmol) was added, and the reaction solution was warmed to room temperature and stirred for 24 h. The resulting solution was concentrated under vacuum. The product was precipitated by the addition of diethyl ether and collected by filtration to produce the title compound **2b** (1.7 g, yield 99%) as a white solid. ESI^+^/MS: 154.1 [M + H]^+^. ^1^H NMR (300 MHz, DMSO-*d*_*6*_) δ 8.26 (d, *J* = 2.2 Hz, 1H), 7.90 (d, *J* = 2.2 Hz, 1H), 7.34 (s, 2H), 3.85 (s, 3H) (Zhou et al., 2015).

#### 4.1.4 3-Aminopyrazine-2-carbohydrazide (**3b**)

To a solution of 450 mg of compound **2b** (3.0 mmol) in 10 ml of ethanol, 3 ml of 85% hydrazine hydrate solution was added at 0°C. The resulting solution was stirred at room temperature for 4 h. The precipitates were filtered under vacuum and rinsed with dichloromethane (3 × 2 ml) and ethanol (3 × 2 ml) to yield the title compound **3b** (108 mg, yield 24%) as a light yellow solid. ESI^+^/MS: 154.1 [M + H]^+^. ^1^H NMR (300 MHz, DMSO-*d*_6_) δ 9.80 (s, 1H), 8.17 (d, *J* = 2.3 Hz, 1H), 7.78 (d, *J* = 2.3 Hz, 1H), 7.47 (s, 2H), 4.50 (s, 2H) (Zhou et al., 2015).

#### 4.1.5 General procedure for the preparation of **4–7**

To a solution of 1 mmol of pyrazine-2-carbohydrazide (**3a**) or 3-aminopyrazine-2-carbohydrazide (**3b**) and 1.1 mmol of 4-chloroquinazoline or 4-chloro-8-methoxyquinazoline in 5 ml of acetonitrile or DMF, 137 µL of triethylamine (1 mmol) was added at 0°C. The resulting solution was stirred overnight at room temperature and then quenched with 20 ml of iced water and 75 ml of ethyl acetate. The organic phase was dried over with anhydrous sodium sulfate and concentrated under vacuum, and the residue was purified through flash chromatography on silica gel with dichloromethane/methanol to afford title compounds **4–7** (yield 44%–58%).

##### 4.1.5.1 Nʹ-(Quinazolin-4-yl)pyrazine-2-carbohydrazide (**4**)

Starting with 138 mg of pyrazine-2-carbohydrazide (**3a**, 1 mmol) and 181 mg of 4-chloroquinazoline (1.1 mmol), 145 mg of the title compound **4** (yield 55%) was obtained as a light yellow solid. ESI^+^/MS: 267.1 [M + H]^+^. ^1^H NMR (300 MHz, DMSO-*d*_*6*_) δ 11.91 (s, 2H), 9.28 (d, *J* = 1.4 Hz, 1H), 8.91 (d, *J* = 2.5 Hz, 1H), 8.78 (dd, *J* = 2.4, 1.5 Hz, 1H), 8.08 (s, 1H), 8.02 (d, *J* = 7.8 Hz, 1H), 7.56 (t, *J* = 7.6 Hz, 1H), 7.37 (t, *J* = 7.5 Hz, 1H), 7.24 (d, *J* = 7.9 Hz, 1H). ^13^C NMR (201 MHz, DMSO-*d*_*6*_) δ 156.84, 148.12, 148.09, 148.02, 145.22, 143.88, 143.81, 136.51, 132.16, 126.54, 124.00, 117.86, 117.07. HRMS (ESI^+^) calcd for C_13_H_10_N_6_O [M + H]^+^ 267.0994, found 267.1016. HPLC purity, 96.6%.

##### 4.1.5.2 3-Amino-Nʹ-(quinazolin-4-yl)pyrazine-2-carbohydrazide (**5**)

Starting with 153 mg of 3-aminopyrazine-2-carbohydrazide (**3b**, 1 mmol) and 181 mg of 4-chloroquinazoline (1.1 mmol), 162 mg of title compound **5** (yield 58%) was obtained as an orange solid. ESI+/MS: 282.1 [M + H]^+^. ^1^H NMR (300 MHz, DMSO-*d*_*6*_) δ 11.91 (s, 2H), 9.28 (d, *J* = 1.4 Hz, 1H), 8.91 (d, *J* = 2.5 Hz, 1H), 8.78 (dd, *J* = 2.4, 1.5 Hz, 1H), 8.08 (s, 1H), 8.02 (d, *J* = 7.8 Hz, 1H), 7.56 (t, *J* = 7.6 Hz, 1H), 7.37 (t, *J* = 7.5 Hz, 1H), 7.24 (d, *J* = 7.9 Hz, 1H). ^13^C NMR (75 MHz, DMSO-*d*_*6*_) δ 161.46, 155.52, 147.67, 147.37, 136.24, 132.04, 131.67, 126.53, 125.97, 124.05, 117.88, 116.84. HRMS (ESI^+^) calcd for C_13_H_11_N_7_O [M + H]^+^ 282.1103, found 282.1133. HPLC purity, 95.5%.

##### 4.1.5.3 Nʹ-(8-Methoxyquinazolin-4-yl)pyrazine-2-carbohydrazide (**6**)

Starting with 138 mg of pyrazine-2-carbohydrazide (**3a**, 1 mmol) and 214 mg of 4-chloro-8-methoxyquinazoline (1.1 mmol), 131 mg of title compound **6** (yield 44%) was obtained as a light yellow solid. ESI^+^/MS: 297.1 [M + H]^+^. ^1^H NMR (300 MHz, DMSO-*d*_*6*_) δ 11.85 (s, 1H), 11.62 (s, 1H), 9.27 (d, *J* = 1.3 Hz, 1H), 8.91 (d, *J* = 2.4 Hz, 1H), 8.84–8.70 (m, 1H), 7.94 (s, 1H), 7.58 (d, *J* = 7.6 Hz, 1H), 7.31 (t, *J* = 8.0 Hz, 1H), 7.21 (d, *J* = 7.6 Hz, 1H), 3.94 (s, 3H). ^13^C NMR (201 MHz, DMSO-*d*_*6*_) δ 160.93, 155.70, 147.09, 146.70, 146.56, 144.22, 142.87, 142.82, 142.72, 142.64, 125.42, 114.19, 111.73, 55.56. HRMS (ESI^+^) calcd for C_14_H_12_N_6_O_2_ [M + H]^+^ 297.1100, found 297.1187. HPLC purity, 95.9%.

##### 4.1.5.4 3-Amino-Nʹ-(8-methoxyquinazolin-4-yl)pyrazine-2-carbohydrazide (**7**)

Starting with 153 mg of 3-aminopyrazine-2-carbohydrazide (**3b**, 1 mmol) and 214 mg of 4-chloro-8-methoxyquinazoline (1.1 mmol), 145 mg of the title compound **7** (yield 47%) was obtained as a yellow solid. ESI^+^/MS: 312.1 [M + H]^+^. ^1^H NMR (300 MHz, DMSO-*d*_*6*_) δ 11.82 (s, 1H), 11.50 (s, 1H), 8.23 (s, 1H), 7.95–7.81 (m, 1H), 7.54 (d, *J* = 7.8 Hz, 1H), 7.29 (t, *J* = 8.0 Hz, 1H), 7.18 (d, *J* = 7.8 Hz, 1H), 3.93 (s, 3H). ^13^C NMR (201 MHz, DMSO-*d*_6_) δ 160.18, 155.73, 147.69, 147.43, 147.35, 147.11, 131.55, 126.59, 126.38, 126.04, 118.64, 115.17, 112.56, 56.60. HRMS (ESI^+^) calcd for C_14_H_13_N_7_O_2_ [M + H]^+^ 312.1209, found 312.1239. HPLC purity, 95.2%.

#### 4.1.6 2-Chloro-N-(pyrazin-2-ylmethyl)quinazolin-4-amine (**9a**)

To a solution of 66 mg of 2,4-dichloroquinazoline (**8a**, 0.33 mmol) in 5 ml of acetonitrile, 96 µL of 1-(pyrazin-2-yl)methanamine hydrochloride (1 mmol) was added at 0°C. The reaction mixture was stirred overnight at room temperature. The precipitate was filtrated, washed with acetonitrile (3 × 2 ml), and purified through flash chromatography on silica gel with dichloromethane/methanol to afford the title compound **9a** (67 mg, 74%) as a light yellow solid. ESI^+^/MS: 272.2 [M + H]^+^. ^1^H NMR (300 MHz, DMSO-*d*_6_) δ 9.42 (t, 1H), 8.73 (d, *J* = 1.0 Hz, 1H), 8.63–8.58 (m, 1H), 8.55 (d, *J* = 2.5 Hz, 1H), 8.33 (d, *J* = 7.9 Hz, 1H), 7.83 (t, *J* = 7.1 Hz, 1H), 7.65 (d, *J* = 8.0 Hz, 1H), 7.58 (t, *J* = 7.6 Hz, 1H), 4.88 (d, *J* = 5.7 Hz, 2H).

#### 4.1.7 2-Chloro-8-methoxy-N-(pyrazin-2-ylmethyl)quinazolin-4-amine (**9b**)

To a solution of 229 mg of 2,4-dichloro-8-methoxyquinazoline (**8b**, 1 mmol) in 10 ml of acetonitrile, 192 µL of 1-(pyrazin-2-yl)methanamine hydrochloride (2 mmol) was added at 0°C. The reaction mixture was stirred overnight at room temperature. The precipitate was filtrated, washed with acetonitrile (3 × 5 ml), and purified through flash chromatography on silica gel with dichloromethane/methanol to afford the title compound **9b** (213 mg, 71%) as a light yellow solid. ESI^+^/MS: 302.1 [M + H]^+^. ^1^H NMR (300 MHz, DMSO-*d*_6_) δ 9.28 (s, 1H), 8.70 (s, 1H), 8.60 (s, 1H), 8.55 (d, *J* = 2.4 Hz, 1H), 7.83 (d, *J* = 8.0 Hz, 1H), 7.49 (t, *J* = 8.1 Hz, 1H), 7.33 (d, *J* = 7.6 Hz, 1H), 4.86 (d, *J* = 5.6 Hz, 2H), 3.90 (s, 3H).

#### 4.1.8 (E)-3-(4-((4-((Pyrazin-2-ylmethyl)amino)quinazolin-2-yl)amino)phenyl)acrylic acid (**10a**)

At room temperature, to a solution of 93 mg of (E)-4-aminocinnamic acid (0.57 mmol) in 5 ml of acetonitrile, 494 mg of Cs_2_CO_3_ (1.52 mmol) was added, and the resulting mixture was refluxed for 20 min. Then, 46 mg of XPhos (0.1 mmol), 89 mg of Pd_2_(dba)_3_ (0.1 mmol), and 137 mg of 2-chloro-N-(pyrazin-2-ylmethyl)quinazolin-4-amine (**9a**, 0.5 mmol) were added in 5 ml of acetonitrile, and the reaction solution was successively refluxed for 48 h. The precipitated solid was filtrated off, washed with acetonitrile (2 × 3 ml), and purified through flash chromatography on silica gel with dichloromethane/methanol to afford **10a** (94 mg, yield 47%) as a brown solid. ESI^+^/MS: 399.2 [M + H]^+^. ^1^H NMR (300 MHz, DMSO-*d*_*6*_) δ 12.39 (s, 1H), 9.26 (d, *J* = 126.3 Hz, 1H), 8.75 (s, 1H), 8.64 (s, 1H), 8.55 (d, *J* = 2.3 Hz, 1H), 8.19 (d, *J* = 8.1 Hz, 1H), 8.14 (s, 1H), 7.82 (s, 1H), 7.79 (s, 1H), 7.67 (t, *J* = 7.5 Hz, 1H), 7.55 (s, 1H), 7.50 (t, *J* = 7.2 Hz, 2H), 7.28 (t, *J* = 7.5 Hz, 1H), 6.36 (d, *J* = 16.0 Hz, 1H), 4.93 (s, 2H). ^13^C NMR (75 MHz, DMSO) δ 168.69, 165.19, 160.68, 160.61, 156.85, 154.73, 151.46, 144.50, 144.07, 144.00, 143.74, 143.65, 133.37, 129.16, 126.91, 125.92, 123.36, 122.55, 118.70, 116.72, 112.18, 44.37. HRMS (ESI^+^) calcd for C_22_H_18_N_6_O_2_ [M + H]^+^ 399.1570, found 399.1609. HPLC purity, 95.3%.

#### 4.1.9 (E)-3-(4-((4-((Pyrazin-2-ylmethyl)amino)quinazolin-2-yl)amino)phenyl)acrylamide (**11a**)

At 0°C, 50 mg of (E)-3-(4-((4-((pyrazin-2-ylmethyl)amino)quinazolin-2-yl)amino)phenyl)acrylic acid (**10a**, 0.13 mmol) was dissolved in 1 ml of SOCl_2_. The reaction solution was stirred at room temperature for 4 h and concentrated under vacuum. The residue was dissolved with 2 ml of THF, and then 4 ml of concentrated ammonia solution was added. The resulting mixture was stirred at room temperature overnight and concentrated again under vacuum. The residue was dissolved in 25 ml of ethyl acetate and washed with a saturated sodium chloride solution (10 ml). The separated organic layer was concentrated, and the residue was purified through flash chromatography on silica gel with petroleum ether/ethyl acetate to give **11a** (15 mg, yield 31%) as a light brown solid. ESI^+^/MS: 398.2 [M + H]^+^. ^1^H NMR (800 MHz, DMSO-*d*_6_) δ 9.13 (d, *J* = 273.8 Hz, 1H), 8.75 (s, 1H), 8.64 (s, 1H), 8.54 (s, 1H), 8.36 (s, 2H), 8.19 (d, *J* = 8.0 Hz, 1H), 7.80 (s, 2H), 7.64 (t, *J* = 7.5 Hz, 1H), 7.46 (dd, *J* = 15.5, 7.0 Hz, 2H), 7.39 (d, *J* = 7.8 Hz, 2H), 7.34 (d, *J* = 15.8 Hz, 1H), 7.25 (t, *J* = 7.4 Hz, 1H), 6.97 (s, 1H), 6.46 (d, *J* = 15.8 Hz, 1H), 4.93 (s, 2H). ^13^C NMR (201 MHz, DM-d6) SO δ 167.60, 165.58, 160.64, 160.59, 156.85, 154.80, 151.54, 144.51, 143.99, 143.61, 143.14, 143.04, 139.79, 133.36, 128.43, 127.33, 125.90, 123.41, 122.46, 119.47, 118.73, 112.12, 44.18. HRMS (ESI^+^) calcd for C_22_H_19_N_7_O [M + H]^+^ 398.1729, found 398.1765. HPLC purity, 95.2%.

#### 4.1.10 (E)-3-(4-((8-Methoxy-4-((pyrazin-2-ylmethyl)amino)quinazolin-2-yl)amino)phenyl)acrylic acid (**10b**)

At room temperature, to a solution of 61 mg of (E)-4-aminocinnamic acid (0.36 mmol) in 5 ml of acetonitrile, 326 mg of Cs_2_CO_3_ (1 mmol) was added, and the resulting mixture was refluxed for 20 min. Then, 30 mg of XPhos (0.06 mmol), 58 mg of Pd_2_(dba)_3_ (0.06 mmol), and 100 mg of 2-chloro-8-methoxy-N-(pyrazin-2-ylmethyl)quinazolin-4-amine (**9b**, 0.33 mmol) were added in 5 ml of acetonitrile, and the reaction solution was successively refluxed for 48 h. The precipitated solid was filtrated off, washed with acetonitrile (2 × 3 ml), and purified through flash chromatography on silica gel with dichloromethane/methanol to afford **10b** (62 mg, yield 43%) as a brown solid. ESI^+^/MS: 429.2 [M + H]+. 1H NMR (300 MHz, DMSO-*d*_6_) δ 12.39 (s, 1H), 9.71 (s, 1H), 8.72 (s, 1H), 8.65 (s, 1H), 8.54 (d, J = 2.2 Hz, 1H), 8.14 (s, 1H), 7.73 (s, 2H), 7.65–7.43 (m, 3H), 7.27 (s, 1H), 6.37 (d, J = 15.9 Hz, 1H), 4.93 (s, 2H), 3.94 (s, 3H). 13C NMR (75 MHz, DMSO-*d*_6_) δ 168.29, 163.41, 160.80, 154.16, 144.54, 144.38, 143.92, 143.78, 129.32, 119.18, 114.98, 56.41, 44.78. HRMS (ESI+) calcd for C_23_H_20_N_6_O_3_ [M + H]+ 429.1675, found 429.1714. HPLC purity, 96.8%.

#### 4.1.11 (E)-3-(4-((8-Methoxy-4-((pyrazin-2-ylmethyl)amino)quinazolin-2-yl)amino)phenyl)acrylamide (**11b**)

At 0°C, 50 mg of (E)-3-(4-((8-methoxy-4-((pyrazin-2-ylmethyl)amino)quinazolin-2-yl)amino)phenyl)acrylic acid (**10b**, 0.12 mmol) was dissolved in 1 ml of SOCl_2_. The reaction solution was stirred at room temperature for 4 h, and concentrated under vacuum. The residue was dissolved with 2 ml of THF, and then 4 ml of concentrated ammonia solution was added. The resulting mixture was stirred at room temperature overnight and concentrated again under vacuum. The residue was dissolved in 25 ml of ethyl acetate and washed with a saturated sodium chloride solution (10 ml). The separated organic layer was concentrated, and the residue was purified through flash chromatography on silica gel with petroleum ether/ethyl acetate to give **11b** (21 mg, yield 42%) as a light brown solid. ESI^+^/MS: 428.2 [M + H]^+^. ^1^H NMR (300 MHz, DMSO-*d*_*6*_) δ 9.54 (s, 1H), 8.72 (s, 1H), 8.64 (s, 1H), 8.54 (s, 1H), 8.14 (s, 1H), 7.76 (s, 1H), 7.37 (d, *J* = 8.1 Hz, 1H), 7.31 (s, 1H), 7.21 (s, 1H), 6.46 (d, *J* = 15.7 Hz, 1H), 4.92 (s, 1H), 3.92 (s, 2H). ^13^C NMR (201 MHz, DMSO-*d*_*6*_) δ 167.59, 167.54, 167.47, 163.46, 160.64, 155.32, 154.53, 152.91, 144.51, 143.88, 143.71, 139.67, 128.49, 127.76, 122.56, 119.63, 119.60, 118.98, 114.86, 113.03, 112.18, 56.21, 44.67. HRMS (ESI^+^) calcd for C_23_H_21_N_7_O_2_ [M + H]^+^ 428.1835, found 428.1877. HPLC purity, 95.2%.

### 4.2 Biological data

#### 4.2.1 Expression and purification of recombinant p97/VCP

His‐tagged human VCP (hVCP) cDNA was amplified by PCR and cloned into PCR‐linearized pET15 b (Merck Millipore, Burlington, MA, United States) using a CloneEZ Kit (Genscript). The pET15 b‐hVCP plasmid was transformed into *Escherichia coli* BL21 (Rosetta; Novagen, Madison, WI, United States), and the transformed bacteria were pre-cultured in LB medium containing kanamycin and chloramphenicol overnight at 37°C. Protein expression was induced with 1 mmol/L isopropyl‐β‐D‐thiogalactopyranoside. His‐tagged hVCP was purified as previously described (Chou et al., 2011). Briefly, the cells were lysed by 500 W sonication, 180 times, 5 s/tine, 5 s intervals. The lysate was centrifuged at 12,000 ×g for 10 min at 4°C, and the resulting supernatant was loaded onto a Ni Sepharose 6 Fast Flow (GE Healthcare) [equilibrium buffer (50 ml, 10 mM Tris-HCl, 0.5 M NaCl (pH 8.0)]. The column was then flushed with wash buffer, and His-tagged p97 was eluted by stepwise application of 10 ml of imidazole solution buffer (15 mM, 60 mM, and 300 mM imidazole in wash buffer). Fractions were collected and analyzed by 12% SDS/PAGE to evaluate purity. Fractions that contained p97 of ≥95% purity were concentrated to 1 mg/ml, exchanged into storage buffer [10 mM Tris-HCl, pH 8.0, 0.5 M NaCl, 300 mM imidazole, 10% glycerol, 2 mM β-mercaptoethanol, and protease inhibitor tablet], and stored at −80°C.

#### 4.2.2 Microscale thermophoresis assay

Fluorescent labeling of His6-p97 was carried out using the RED-tris-NTA labeling kit (Catalog No. MO-L011, NanoTemper Technologies) on a NanoTemper Monolith NT.115 following the manufacturer’s protocol. In brief, His6-p97 protein was diluted into 200 nM with PBST (0.05% Tween 20 in PBS), and red-NHS dye was diluted into 100 nM with PBST. Then, His6-p97 and RED-tris-NTA dye were mixed in a 1:1 volumetric ratio and incubated at room temperature for 30 min. Next, compounds were titrated in 1% DMSO starting at 200 μM down to 0.0061 μM. Then 10 μL of compounds were mixed at different concentrations with 10 μL of dye-labeled His6-p97. Subsequently, well-mixed solutions containing compounds and dye-labeled His6-p97 were loaded into capillaries (Catalog No. MO-K025, NanoTemper Technologies), which were placed in slots 1–16 on the capillary rack. Then, MST experiments were carried out at an ambient temperature, and the instrument parameters were set to 20% LED power and medium MST power. The equilibrium dissociation constants were derived from the M.O. Affinity Analysis software using the *K*_d_ model based on four independently titrated experiments per condition.

#### 4.2.3 ATPase analysis

Protein concentrations were first normalized using a Pierce BCA protein assay kit (Thermo Fisher), and a standard series was prepared with the BSA provided (2 mg/ml–25 μg/μL). Twenty-five microliters of each standard and unknown sample was added to a 96-well plate in triplicate, and 200 μL of working reagent was added to each well and prepared according to the manufacturer’s guidelines. The plate was covered and incubated at 37°C for 30 min, the plate was cooled to room temperature (RT), and the absorbance at 562 nm was measured for each well.

The ATPase activity was examined using the ADP-Glo kinase assay protocol (Promega) described previously (Anderson et al., 2015). The protein solution was diluted to 20 nM of p97 with 20 μM ATP in triplicate in 5 μL of 1× Kinase Reaction Buffer A (40 mM Tris, pH 7.5, 20 mM MgCl_2_, 0.1 mg/mL BSA) and incubated at 37°C for 30 min. After incubation, the solutions were equilibrated to RT for 5 min, and 5 μL of ADP-Glo reagent was added to stop the reaction and consume the remaining ATP. The solution was incubated for 40 min at RT. Ten microliters of kinase detection reagent was added and incubated for 30 min. The sample’s luminescence was recorded using a multimode microplate reader (SMP7, MD). Each sample was averaged across the three measurements and plotted as mean ± standard deviation.

#### 4.2.4 Cell culture

A549, HCT116, s180, K562, RPMI-8226, HepG2, and NCM460 cells (purchased from KeyGen Biotech Co., Ltd.) were cultured in RPMI-1640 medium or DMEM (KeyGen Biotech Co., Ltd., KGM31800-500, KGM12800-500). The medium was supplemented with 10% fetal bovine serum (FBS) (Hyclone, SH30084.03). Cells were cultured in an incubator containing 5% CO_2_ at 37°C.

#### 4.2.5 Cell proliferation assay

Antiproliferative activity was measured using the Cell-Counting Kit 8 (PBM, 230,801) according to the manufacturer’s protocol. RPMI-1640 or DMEM containing 10% FBS and 1% penicillin/streptomycin was used as the cell viability assay medium. Cells were seeded at 3,000–4,000 cells per well in a 96-well plate to test the antiproliferative activity of target compounds. Cells were treated with the compounds or positive controls 6 h after seeding (twofold dilution, eight concentrations). After 48 h of treatment, cells were incubated with a 10% CCK-8 solution at 37°C for 1 h or 0.5% MTT solution at 37°C for 4 h, and IC_50_ values were calculated using the percentage of growth of treated cells *versus* the DMSO control. The results were analyzed using GraphPad Prism 7.0.

#### 4.2.6 Flow cytometry

The cell cycle kit (YEASEN, 40301ES60) and apoptosis detection kit were purchased from (Invitrogen, 88-8007). The assay was performed as recommended by the manufacturer. Briefly, the HCT116 cells were seeded in 6-well plates (2 × 10^6^ cells/well) and allowed to adhere for 4 hours. Then, each well was treated with DMSO (0.5% v/v) or compounds for 12 h. For the apoptosis experiment, at the end of treatment, the cells were washed twice with cold PBS and then harvested for staining with annexin V in binding buffer for 15 min in the dark at room temperature. The cells were washed in binding buffer and then incubated with propidium iodide. For the cell cycle experiment, at the end of treatment, the cells were washed twice with cold PBS, fixed with 70% cold ethanol, and then harvested for staining with propidium iodide (containing 2% RNaseA) for 30 min in the dark at room temperature. The samples were measured using flow cytometry (Beckman, cytoFLEX) within 4 h and analyzed using CytExpert (V 2.4.0.28).

#### 4.2.7 Western blot

HCT116 cells were plated into 6-well plates (2 × 10^6^ cells/well) when they were 70%–80% confluent. DMSO (0.5% v/v) or compounds were added, and cells were harvested after 8 h of treatment and washed three times with phosphate-buffered saline (PBS, pH 7.4). Cytoplasmic fractions were isolated from the cell pellets using a protein extraction kit (GenePool/GPP 1815). After determining protein concentration using Bradford reagent (Bio-Rad), 90 µg of samples was loaded on 5% concentrated gel and 12%–15% separating gel using the CAVOY system (MP-8001). Proteins were transferred to nitrocellulose membranes using the CAVOY system (MP-3030). Primary antibodies used were K48 Ubiquitin (CST/8081), p97 (Abcam/ab109240), p62 (CST/5114), ATF4 (CST/11815), NF-κB p65 (CST/8242), GAPDH (Abcam/ab181602), CHOP (CST/2895), and LC3 (CST/4108). The secondary antibodies used were goat anti-mouse IgG (HRP, Abcam/ab6789). ECL Plus Western Blot Kit (pg) (GenePool/GPP 1824) and ChemiDoc MP Imaging System (Bio-Rad) were used to image the blots.

#### 4.2.8 *In vivo* anti-tumor assay on s180 mouse model

The assessments described here were performed based on a protocol reviewed and approved by the ethics committee of Capital Medical University (AEEI-2021-202). The committee confirms that the welfare of the animals was maintained in accordance with the requirements of the animal welfare act and with the guide for the care and use of laboratory animals. In *in vivo* anti-tumor assay of the s180 mouse model in this evaluation, ICR mice (male, 22 ± 2 g, commercially provided by Capital Medical University) were used and maintained with a natural day/night cycle. For initiation of subcutaneous tumors, s180 ascites tumor cells were obtained from the tumor-bearing mice that were serially transplanted once per week. To implant subcutaneous tumors, 0.2 ml of NS containing 2 × 10^6^ viable tumor cells was injected under the skin at the right armpit. After 24 h of implantation, the mice were randomly divided into seven groups (12 mice/group) and treated for 10 consecutive days, i.e., compound **6** (oral administration: 90 μmol/kg/day), DBeQ and CB5083 (oral administration: 90 μmol/kg/day, positive control), and 0.5% CMC-Na (oral administration, vehicle control). The mice were weighed, and the tumor volume was measured daily. All mice were weighed 24 h after the last administration, sacrificed by diethyl ether anesthesia, and collected 1 ml of fresh blood. Then, the tumors and organs were dissected and weighed immediately. Tumor volumes were calculated as follows: V (cm^3^) = length (cm) × width (cm)^2^ × π/6.

#### 4.2.9 *In vivo* p97 expression measurement

After the anti-tumor study, 1 ml of fresh blood of the anesthetized mice was collected into centrifuge tubes containing 0.1 ml of 3.8% sodium citrate. Blood samples were centrifuged (1,811 g, 15 min, 4°C) immediately to prepare plasma samples. The tumors were homogenated and washed using cold PBS three times. The precipitate was lysed by PIRA solution containing protease inhibitor and phosphatase inhibitor according to the protocol. Then, the mixture was centrifuged (12,000 rpm, 10 min, 4°C). The supernatant was obtained for the assay. p97 expression levels in the blood and the tumor were measured using enzyme-linked immunosorbent assay (ELISA) according to the instruction of the p97 ELISA kit (FANKEWEI, F31209-A, China). The absorbance at 450 nm of wavelength was measured with a SpectraMax id5 Absorbance Microplate Reader, and the concentration of p97 in the sample was determined using the p97 standard curve. Data were expressed as mean ± standard deviation (SD) ng/ml. The statistical analysis of the data was done using an ANOVA statistical test, with a statistical significance level of *p*-value less than 0.05 (*p* < 0.05).

#### 4.2.10 Myelosuppressive toxicity evaluation

To estimate the toxicity of compound **6** to marrow, markers including white blood cell count (WBC) or leukocytes, absolute lymphocyte count (ALC), absolute neutrophil count (ANC), platelet count (PLT), and red blood cell count (RBC) of s180 mice and normal mice were tested using a blood-cell counter (URIT, China) and related detection kit (URIT 5D 11Vet).

## Data Availability

The raw data supporting the conclusion of this article will be made available by the authors, without undue reservation.
