# Supplementary figures and images for: Discovery of novel benzylquinazoline molecules as p97/VCP inhibitors
Source: Front Pharmacol. 2023 Jun 14;14:1209060. doi: 10.3389/fphar.2023.1209060 (PMC10300352; doi:10.3389/fphar.2023.1209060)

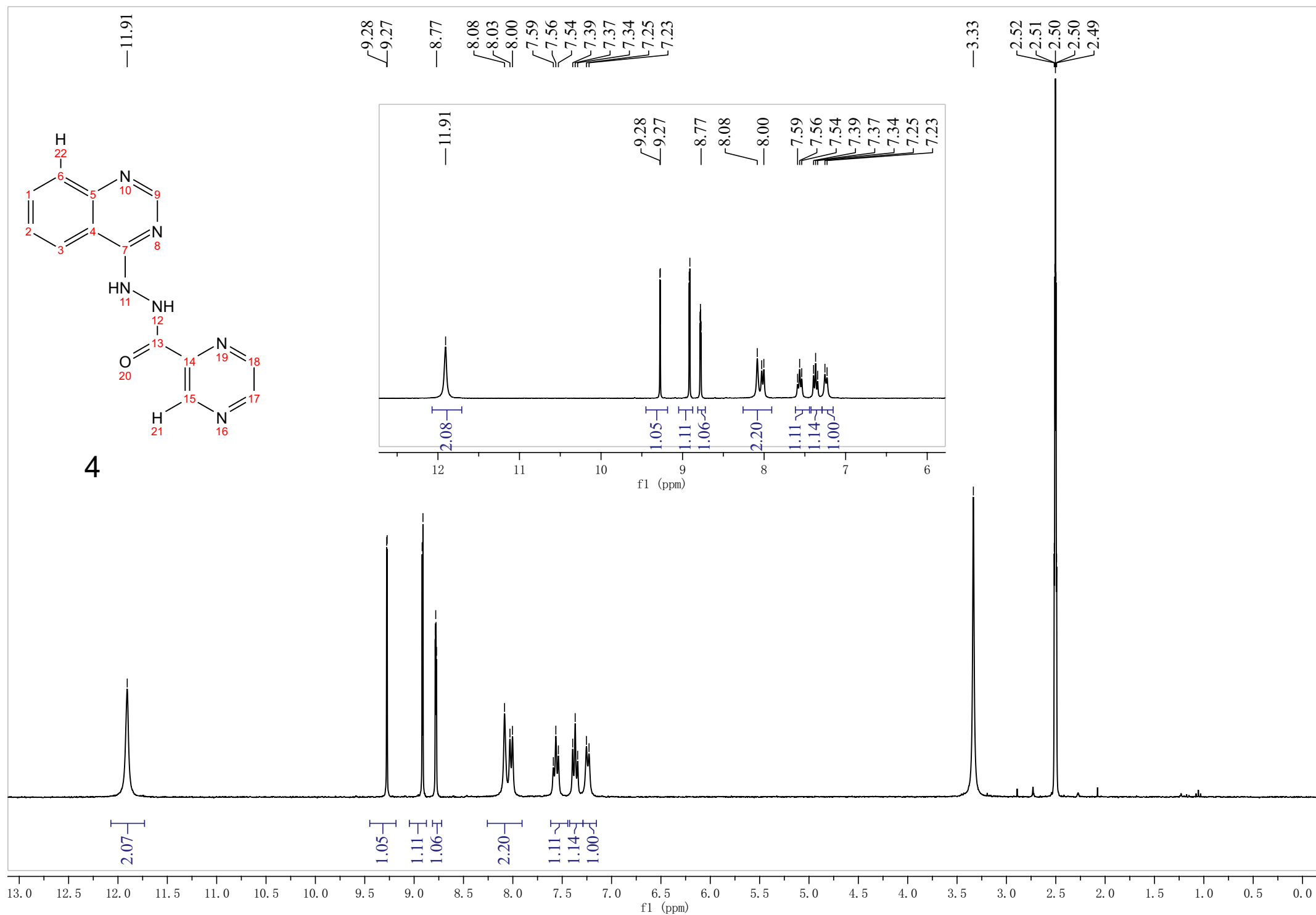

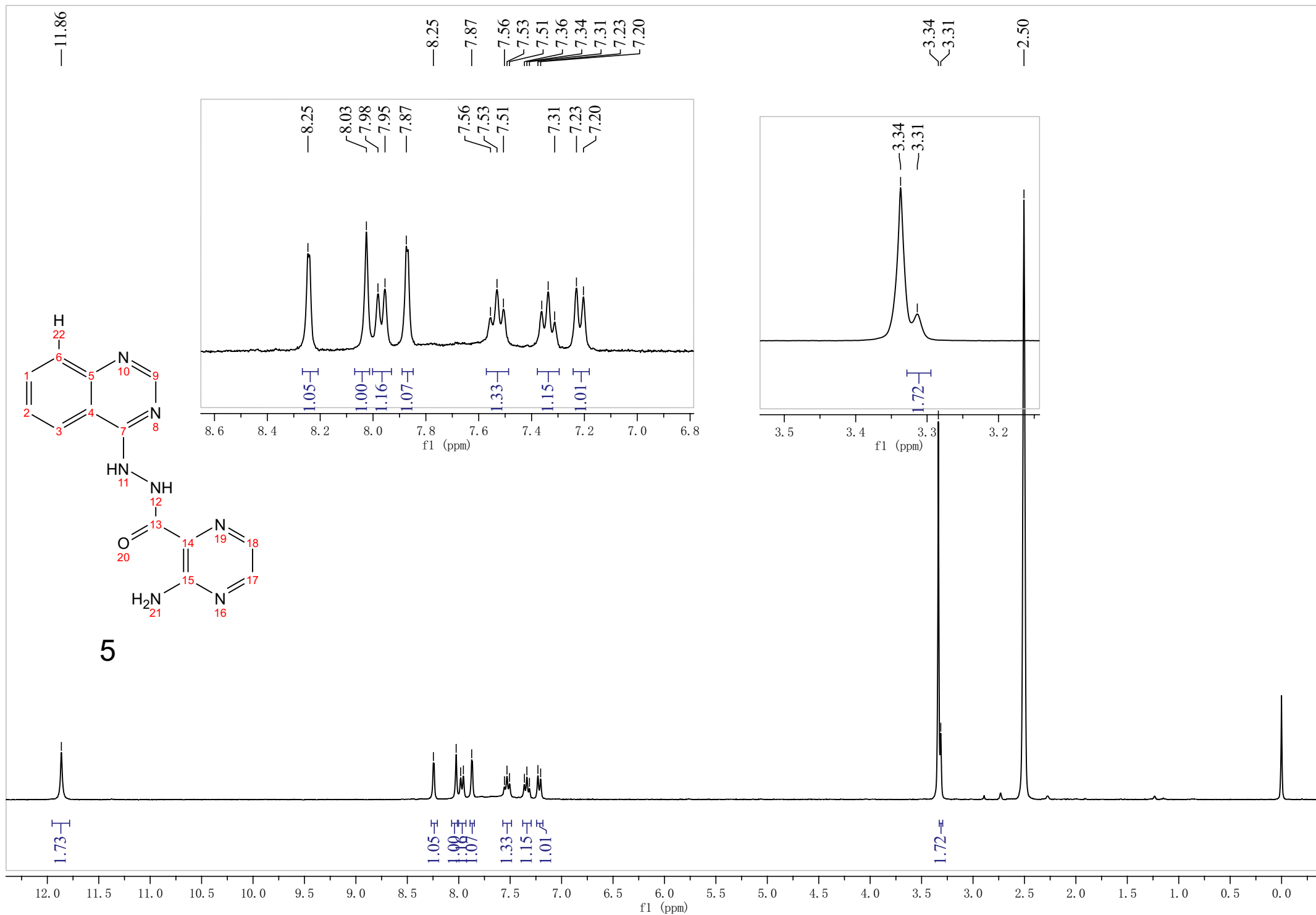

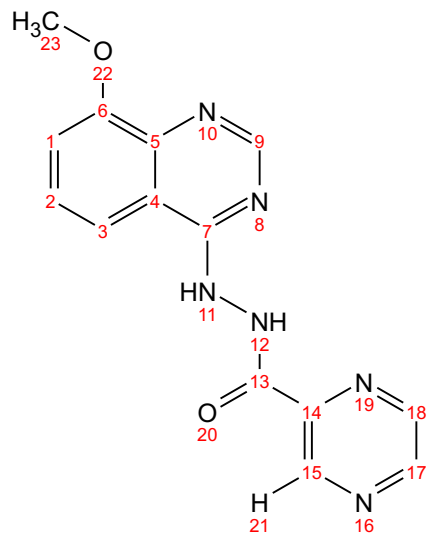

6

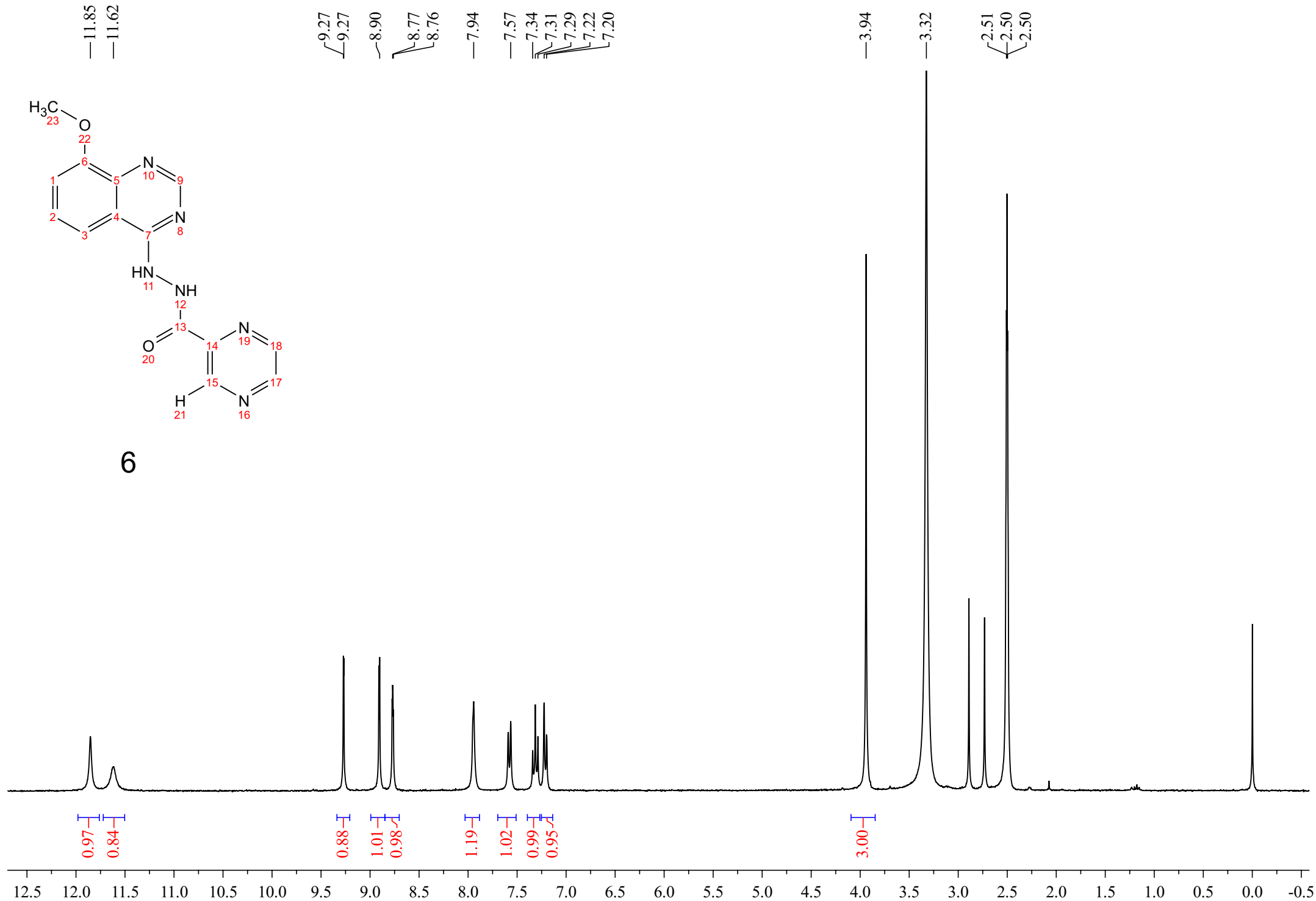

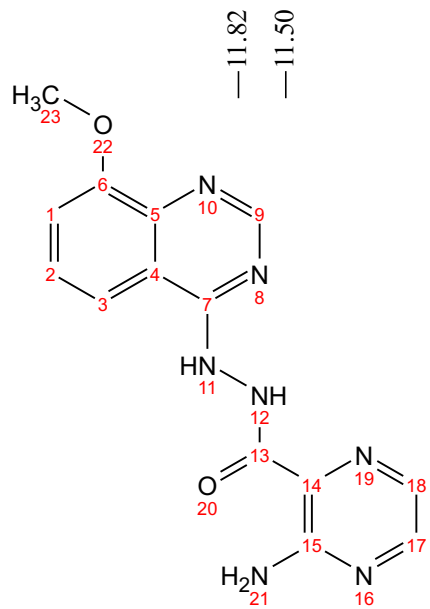

7

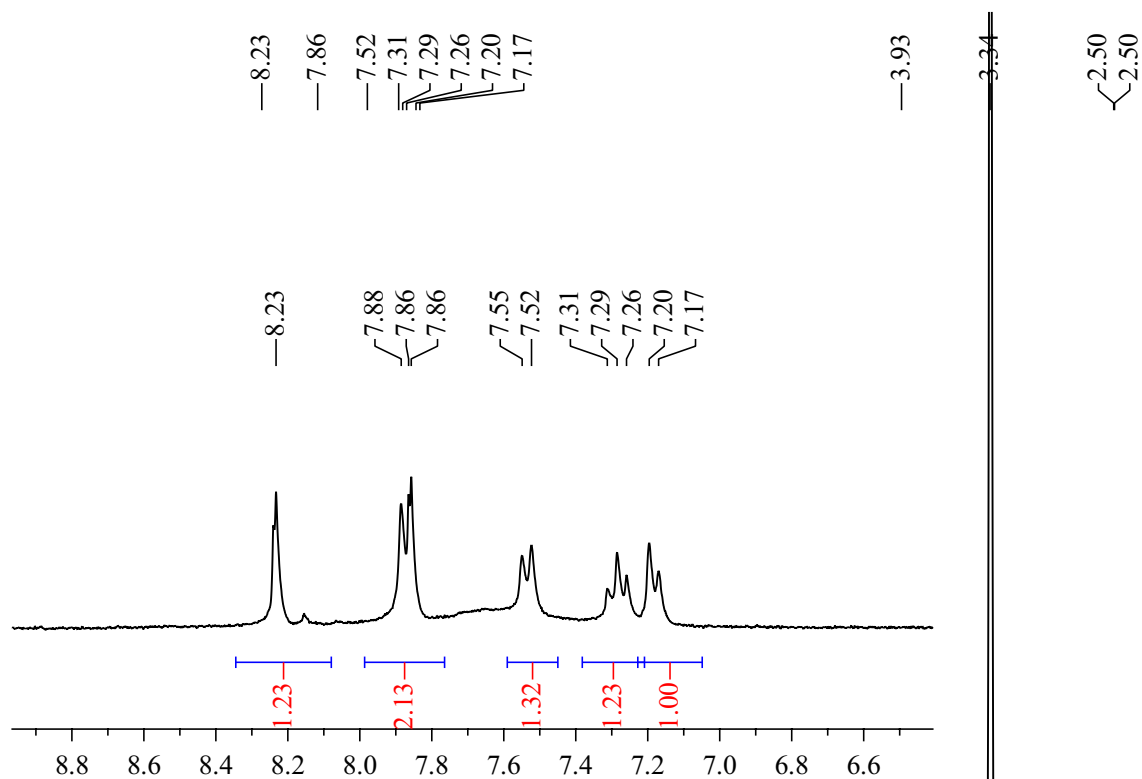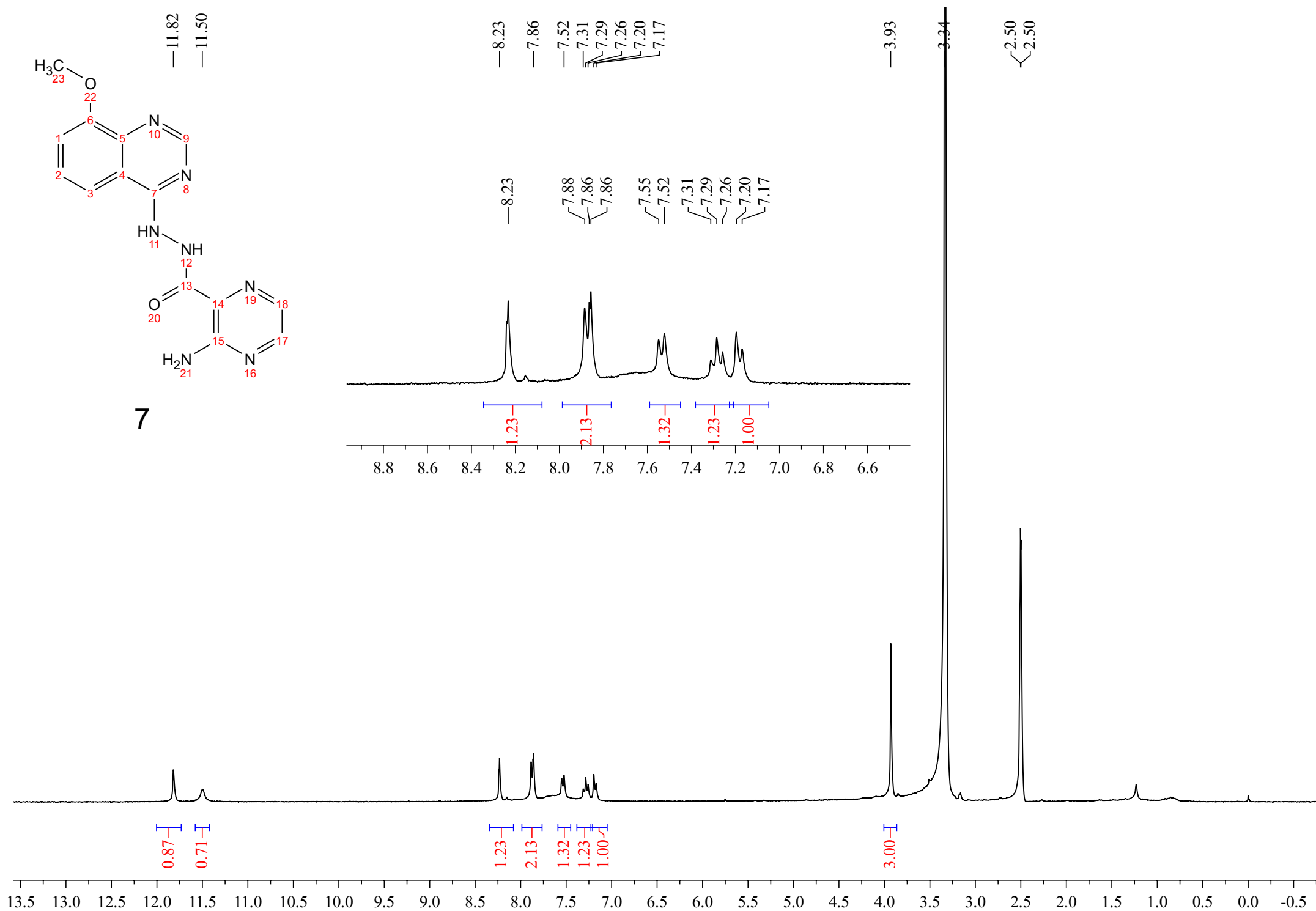

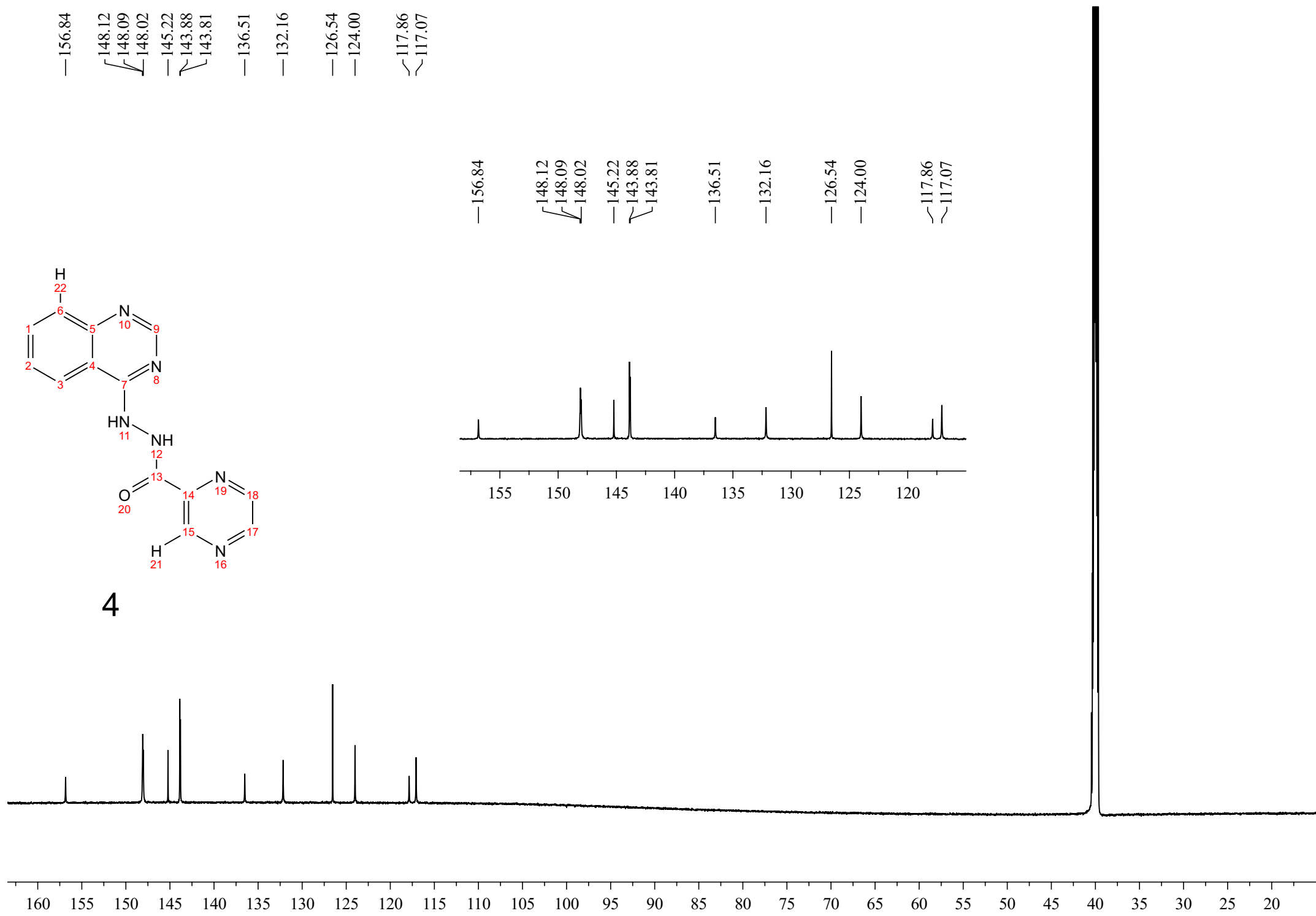

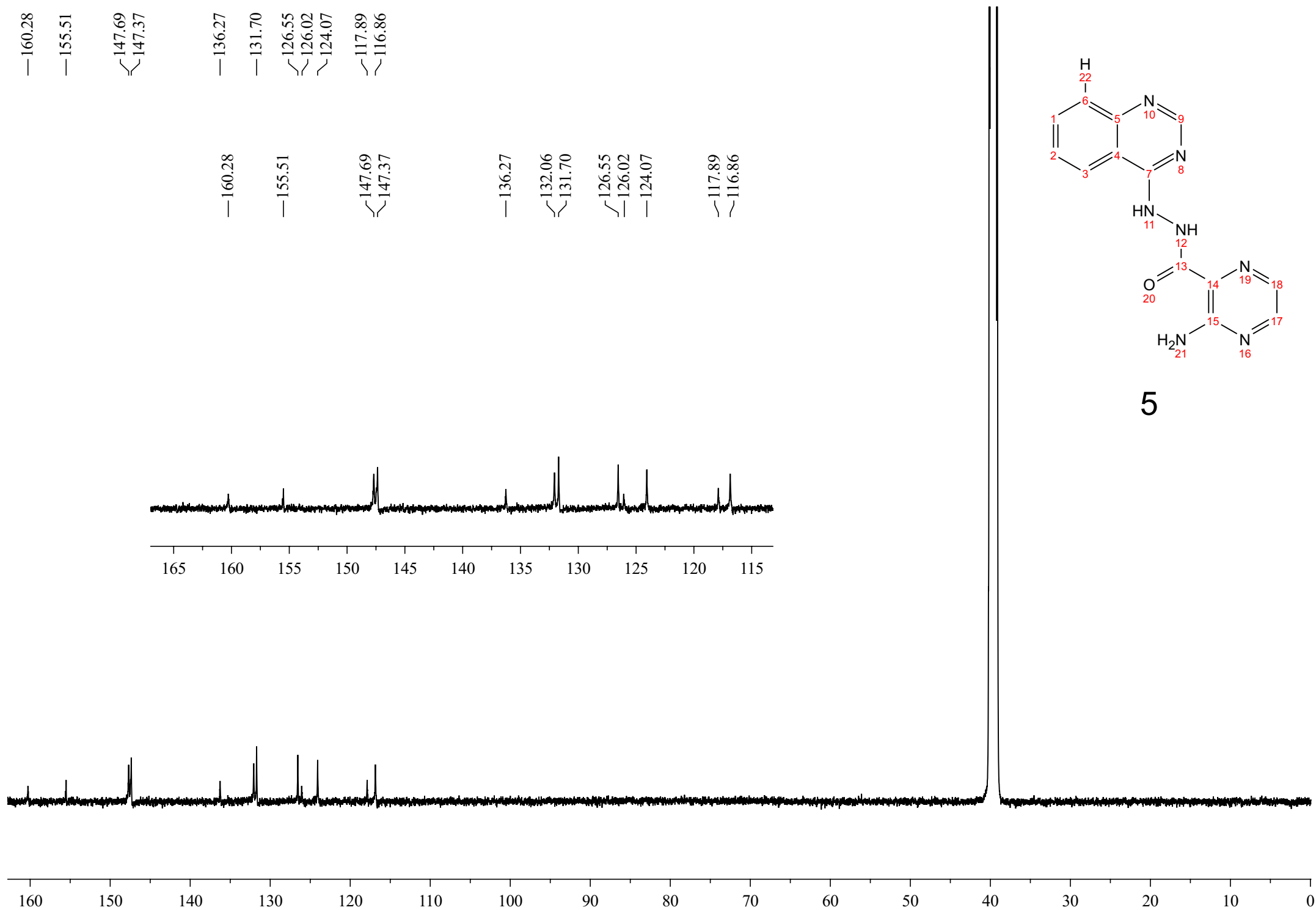

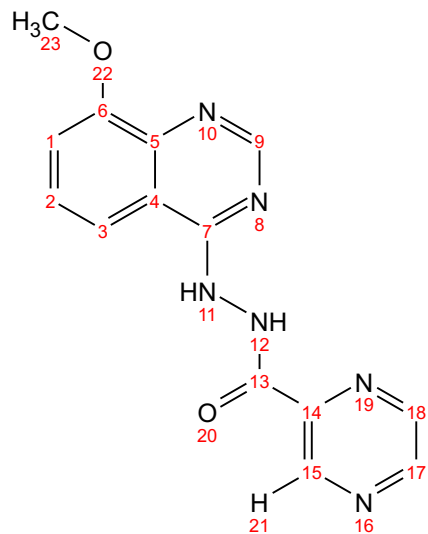

6

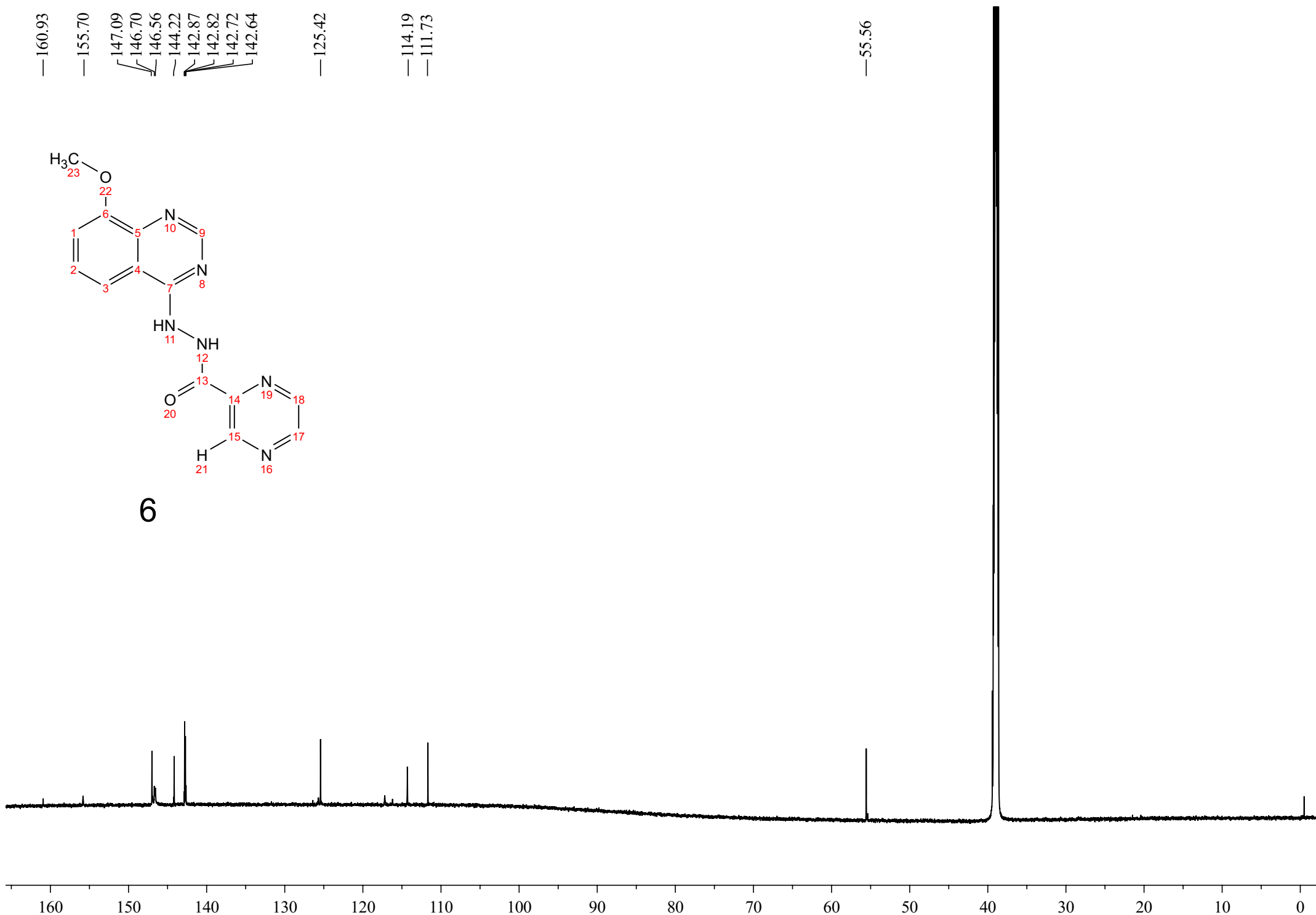

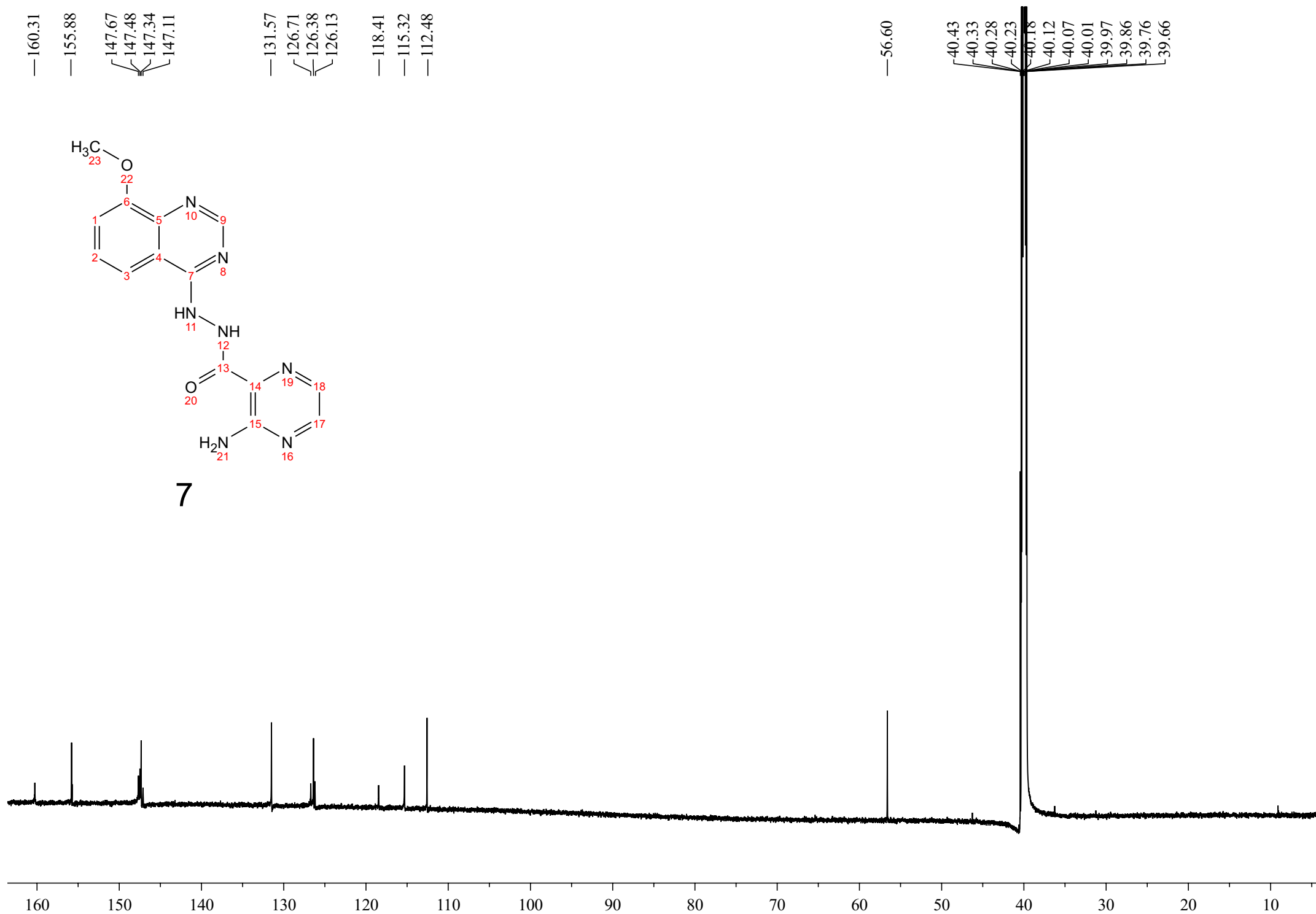

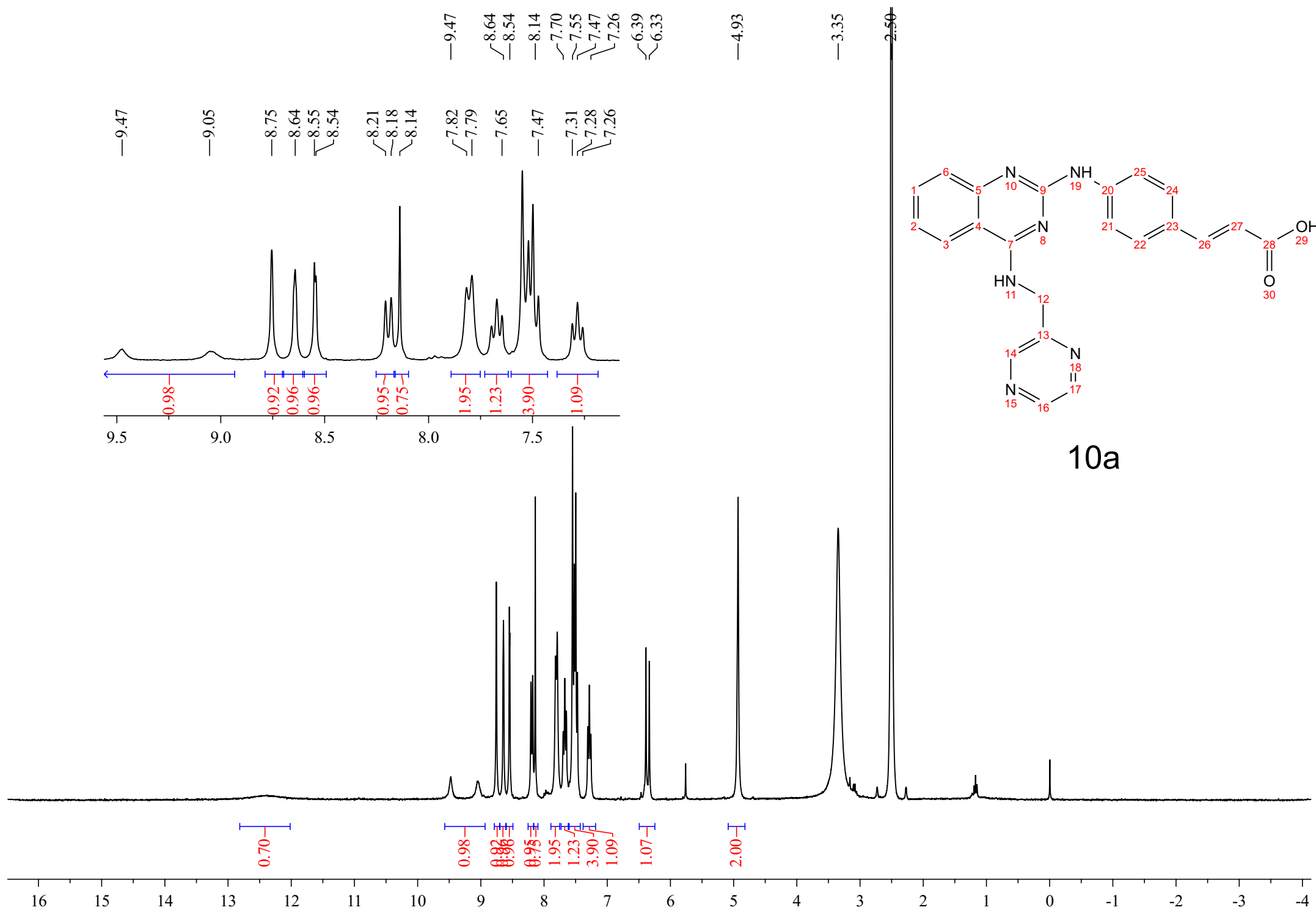

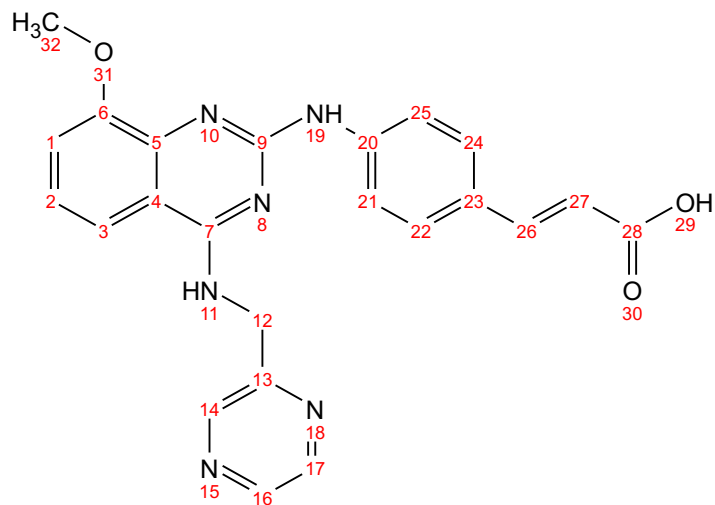

10b

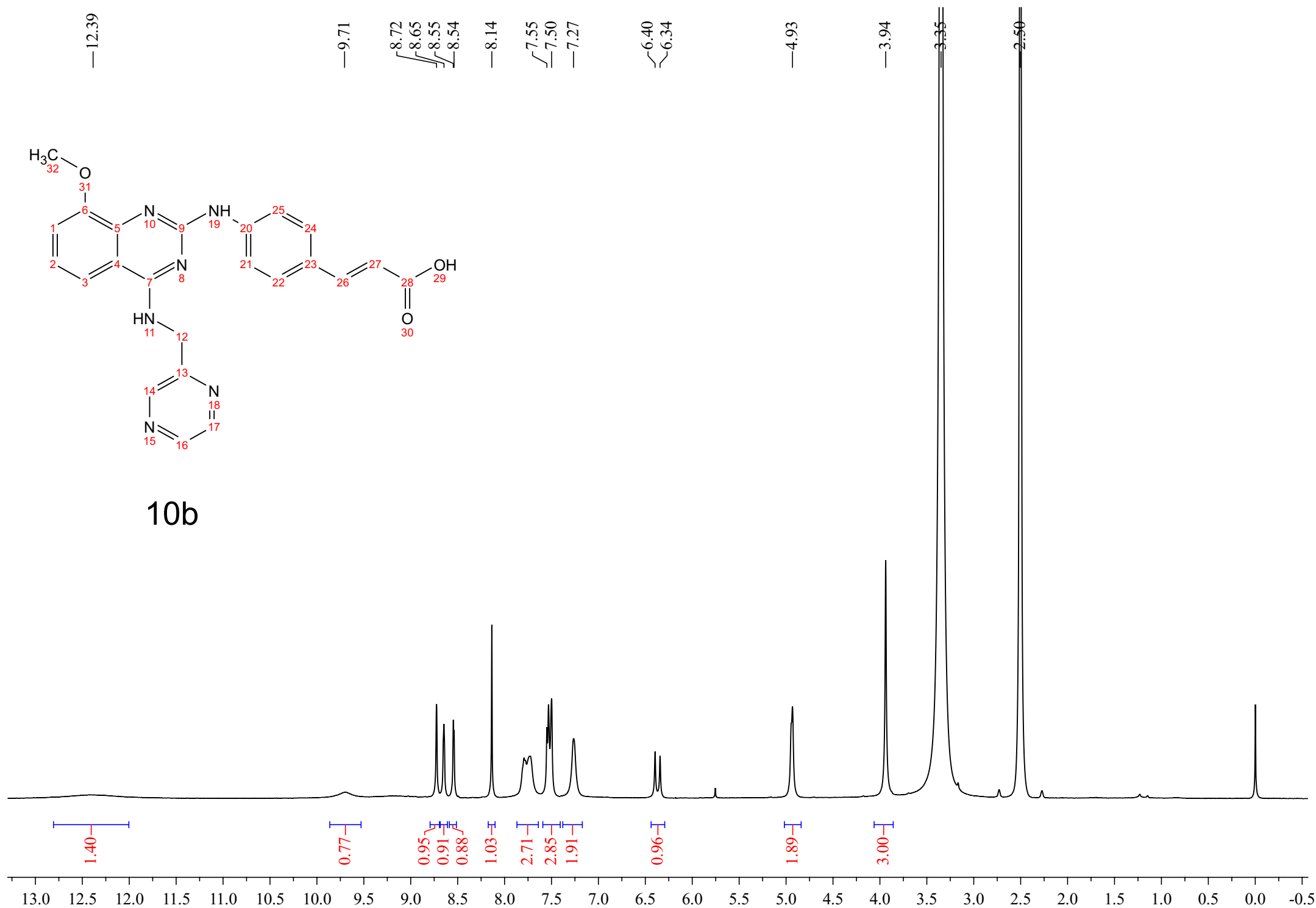

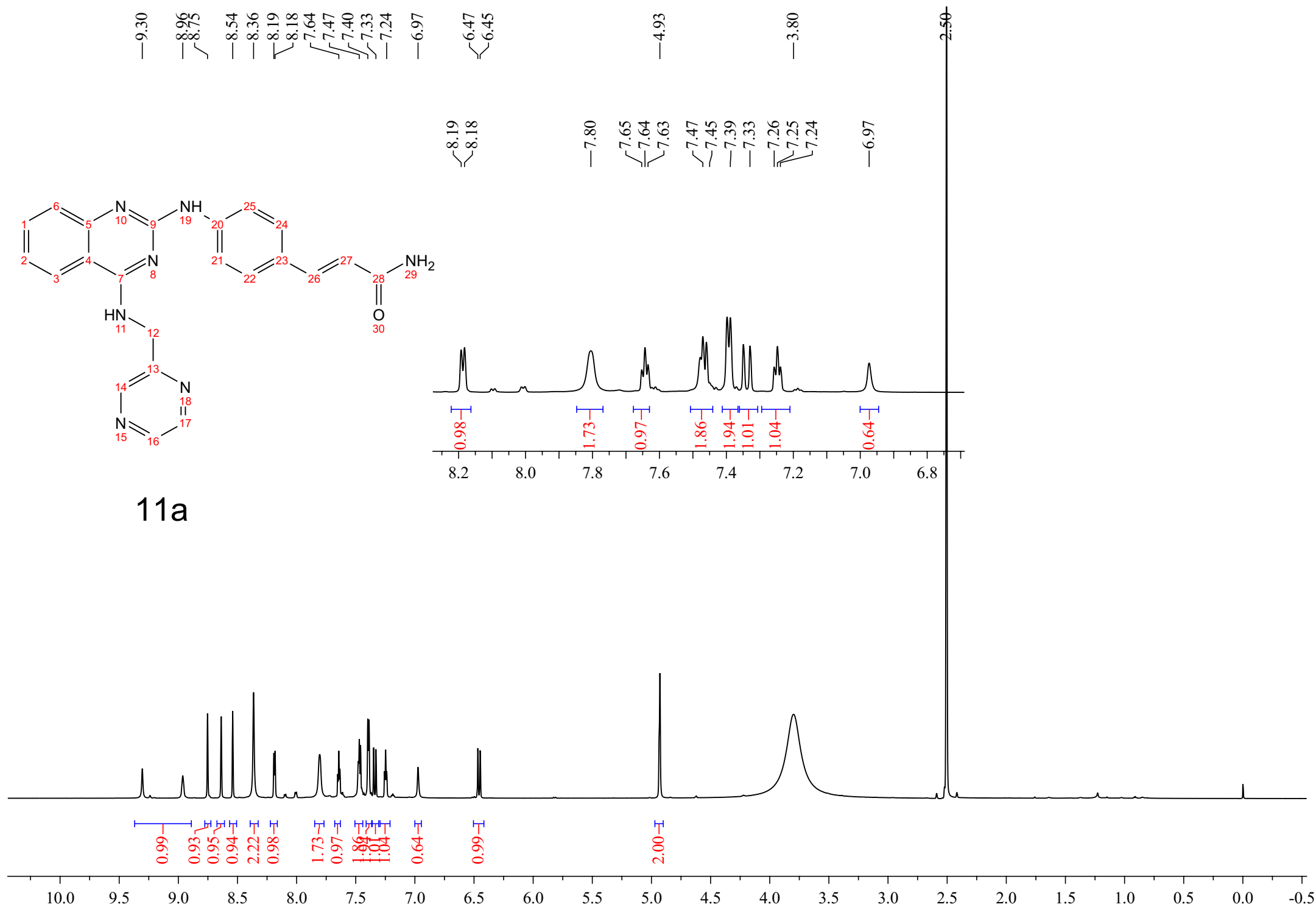

11b

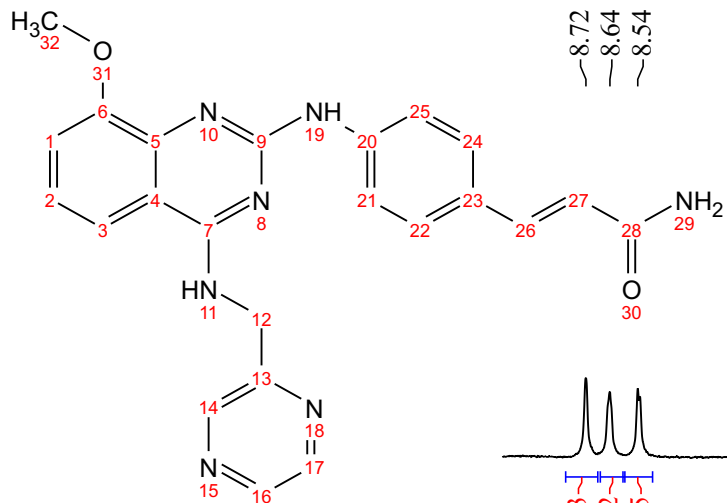

— 9.54

~ 8.72  
~ 8.64  
~ 8.54

— 8.14

— 7.76  
~ 7.39  
~ 7.36  
~ 7.31  
~ 7.21

~ 6.49  
~ 6.43

— 4.92

— 3.92

3.32

2.50

~ 8.72  
~ 8.64  
~ 8.54

— 8.14

— 7.76

~ 7.39  
~ 7.36  
~ 7.31  
~ 7.21

~ 6.49  
~ 6.43

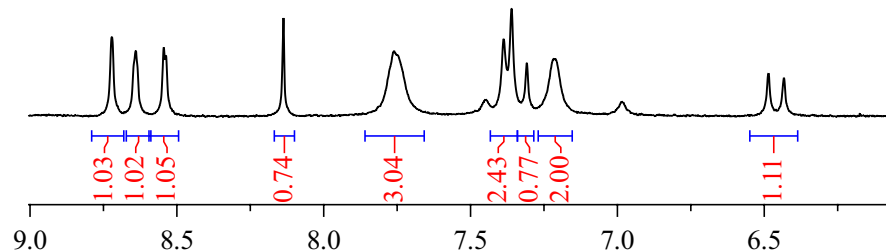

0.63

1.03  
1.02  
1.05

0.74

3.04

2.43  
0.77  
2.00

1.11

2.06

3.00

11.0 10.5 10.0 9.5 9.0 8.5 8.0 7.5 7.0 6.5 6.0 5.5 5.0 4.5 4.0 3.5 3.0 2.5 2.0 1.5 1.0 0.5 0.0 -0.5

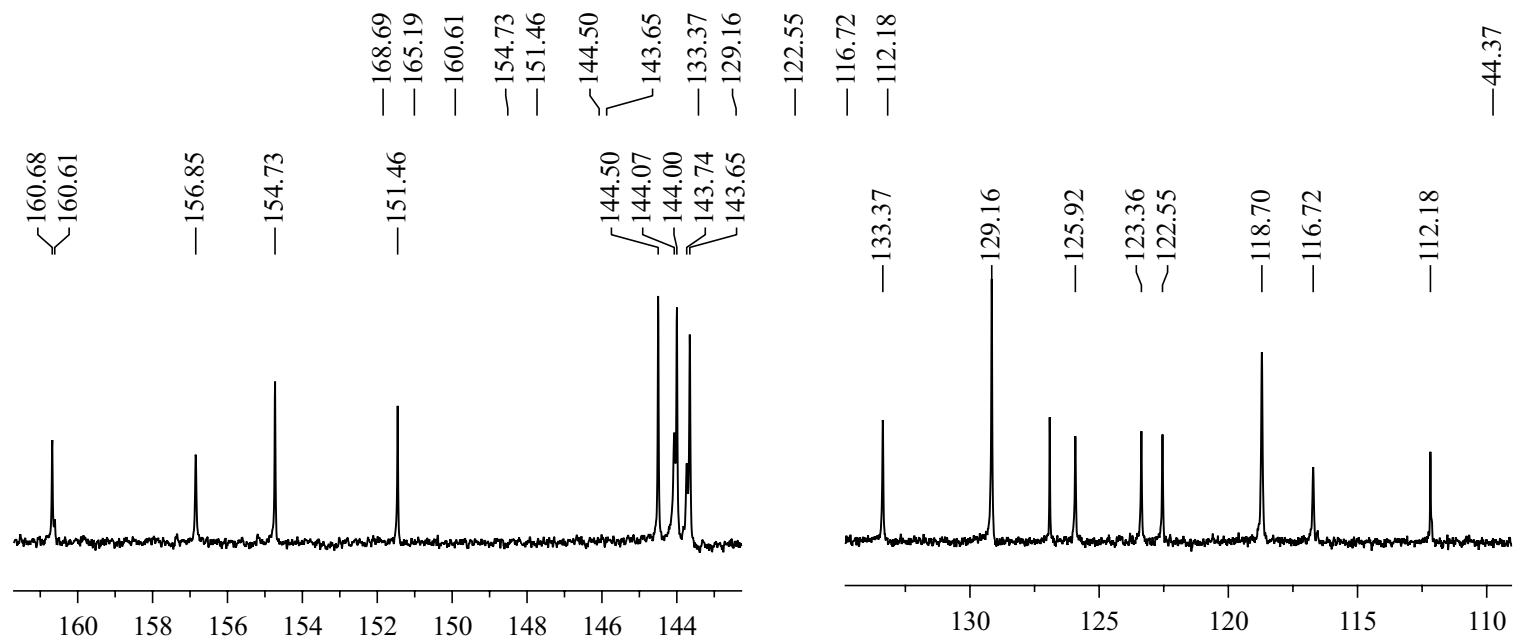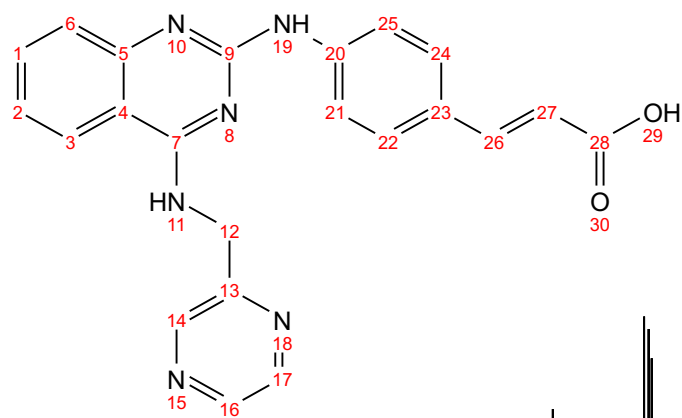

10a

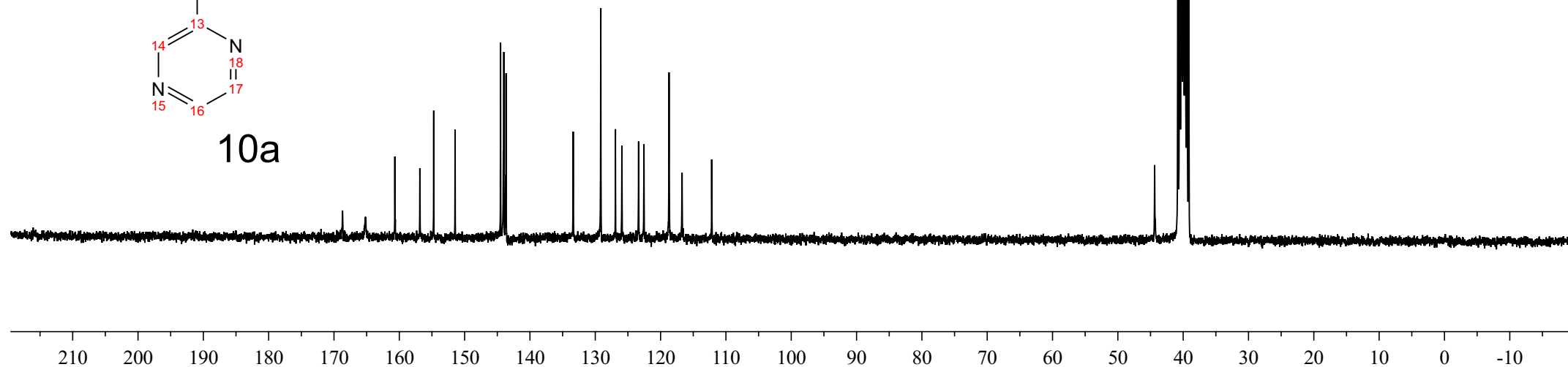

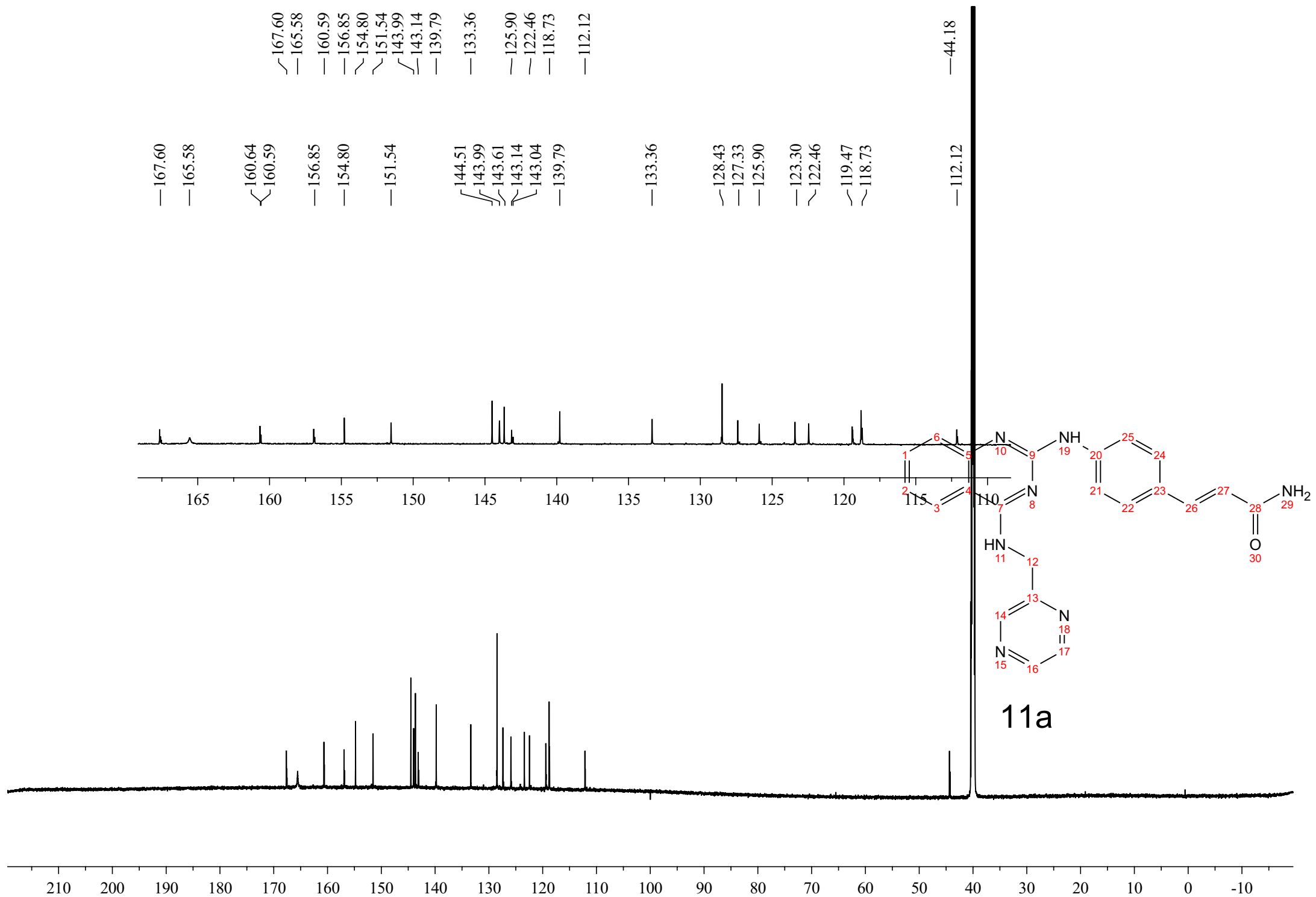

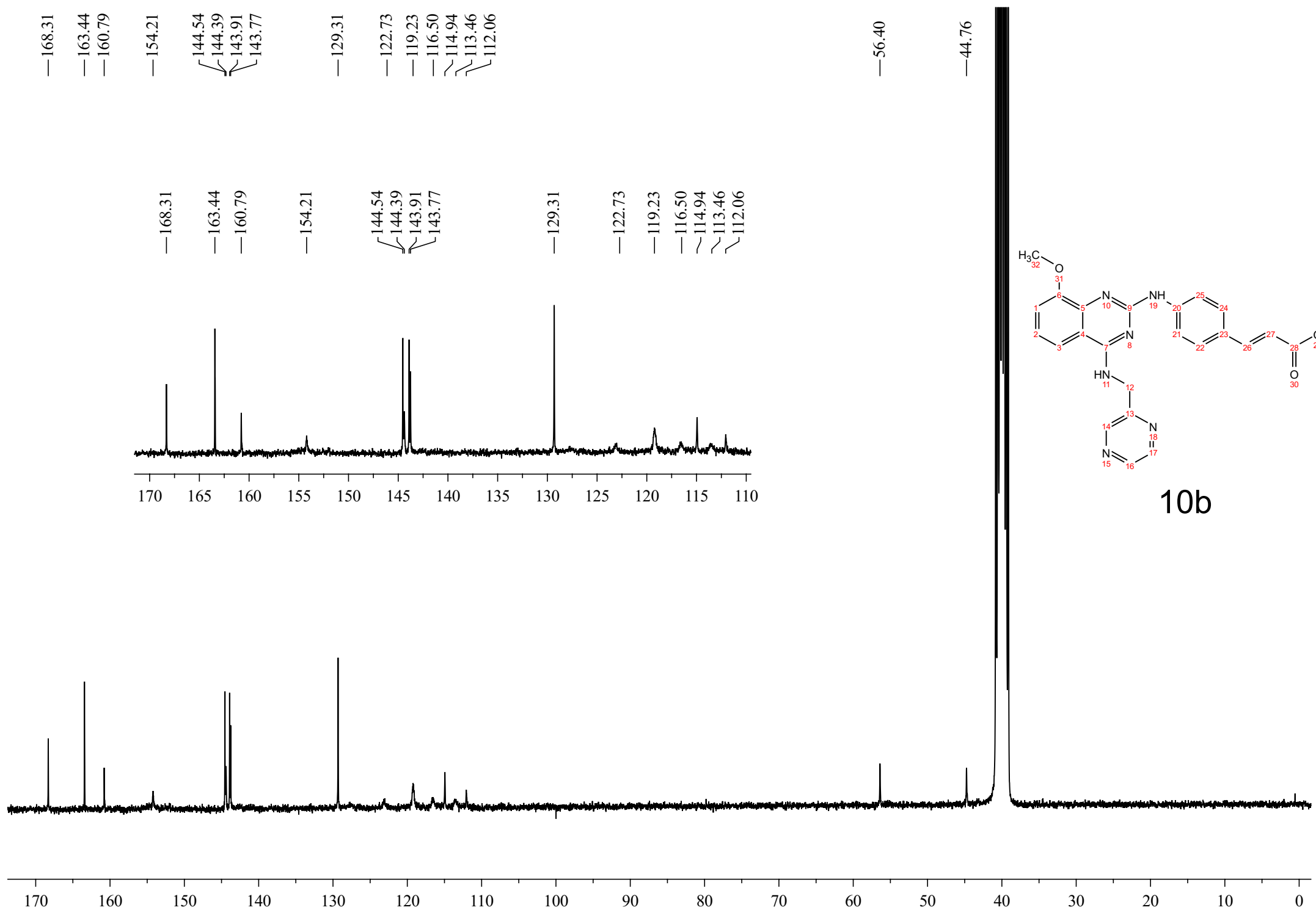

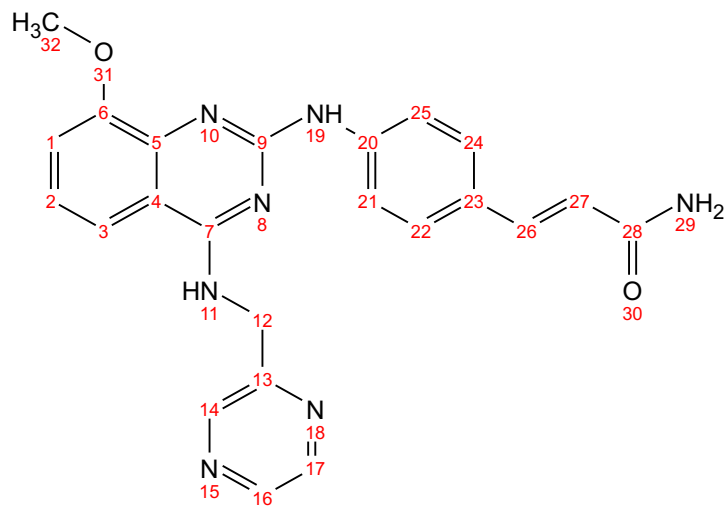

11b

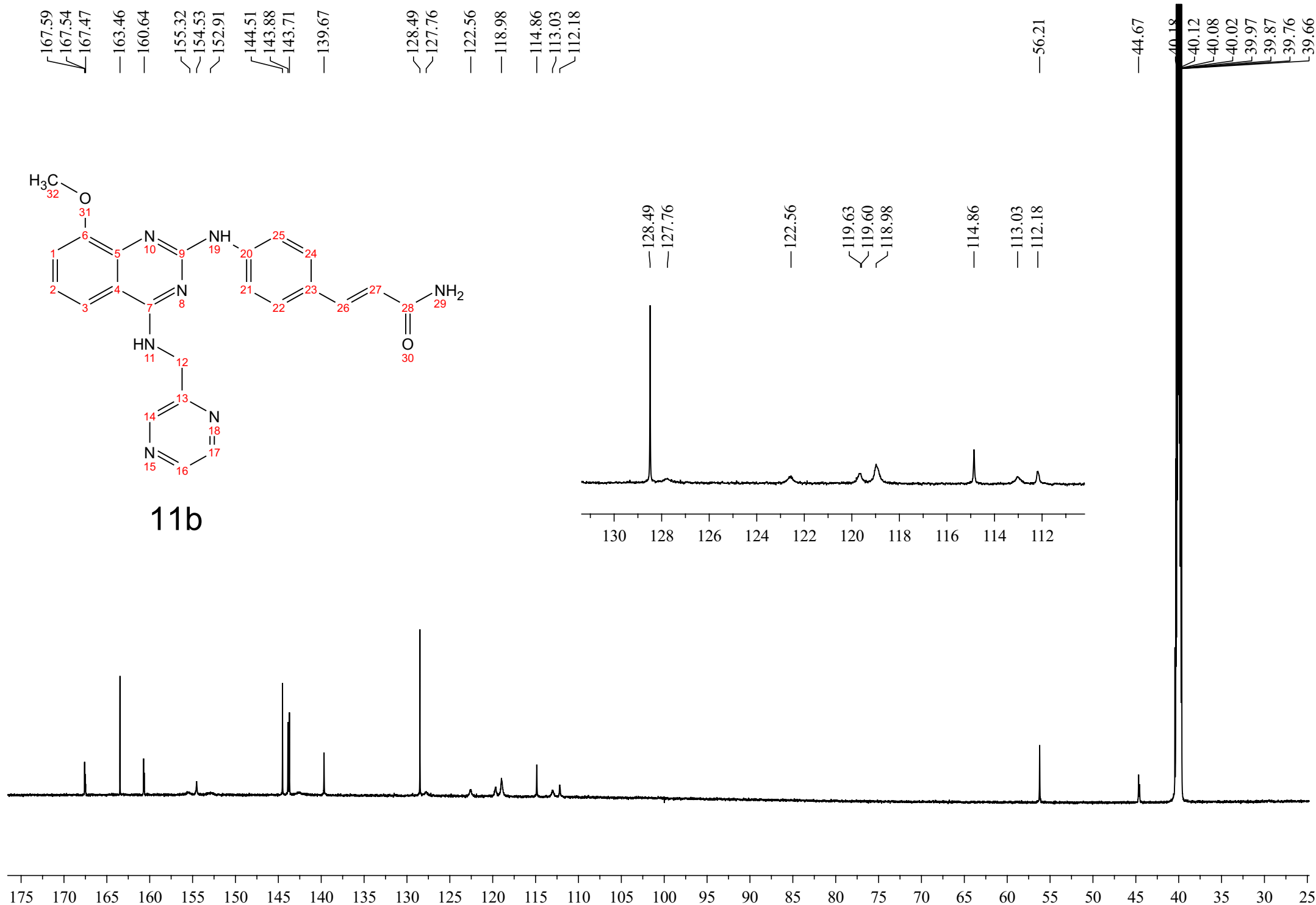

Supplement: Supplementary file 1 [file DataSheet1.PDF]
